# Supplementary material for: The microbiome profiling of fungivorous black tinder fungus beetle Bolitophagus reticulatus reveals the insight into bacterial communities associated with larvae and adults
Source: PeerJ. 2019 May 7;7:e6852. doi: 10.7717/peerj.6852 (PMC6510215; doi:10.7717/peerj.6852)
Supplement: Table S1 [file peerj-07-6852-s001.docx]

Table S1. Comparison the results field observation of numer of *Fomes fomentarius* fruiting bodies healthy and inhabited by mycetophagous beetles, growing on birch and beech trunks.

| **Beech *Fagus*** | | | | **Birch *Betula*** | | | |
| --- | --- | --- | --- | --- | --- | --- | --- |
| **Trunk of *Fagus*** | **Healthy fungi** | **Inhabited fungi** | **amount**  **/trunk** | **Trunk of *Betula*** | **Healthy fungi** | **Inhabited fungi** | **amount**  **/trunk** |
| *F*1 | 15 | 4 | 19 | *B*1 | 2 | 12 | 14 |
| *F*2 | 24 | 12 | 36 | *B*2 | 9 | 9 | 18 |
| *F*3 | 17 | 4 | 21 | *B*3 | 4 | 17 | 21 |
| *F*4 | 16 | 7 | 23 | *B*4 | 7 | 6 | 13 |
| amount | 72 | 27 | 99 | amount | 22 | 44 | 66 |
