# Supplementary material for: The microbiome profiling of fungivorous black tinder fungus beetle Bolitophagus reticulatus reveals the insight into bacterial communities associated with larvae and adults
Source: PeerJ. 2019 May 7;7:e6852. doi: 10.7717/peerj.6852 (PMC6510215; doi:10.7717/peerj.6852)
Supplement: Data S1 — The first level represents the kingdom, the second level represents all phyla present in a particular sample; subsequent next levels represent the class, order, family and genus. [file peerj-07-6852-s003.zip › Supplemental_Data_S1/Im-Betula-1.html]

Javascript must be enabled to view this page.

magnitude

 .999999999999888

 0

 0

 0

 0

 0

 0

 .999999999999888

 5.59151208466E-05

 5.59151208466E-05

 5.59151208466E-05

 5.59151208466E-05

 5.59151208466E-05

 8.23489942267483E-02

 8.23489942267483E-02

 7.66316731201917E-02

 7.66316731201917E-02

 .000517214867831

 .000181724142751

 .0600668185694

 1.39787802116E-05

 8.38726812698E-05

 .00016774536254

 .00349469505291

 .000125809021905

 .00728294449026

 .00466891259069

 2.79575604233E-05

 5.7173211065566E-03

 5.7173211065566E-03

 .00566140598571

 5.59151208466E-05

 0

 0

 0

 0

 0

 0

 0

 0

 0

 0

 0

 0

 0

 0

 0

 0

 0

 0

 0

 0

 0

 0

 0

 0

 0

 0

 0

 0

 0

 0

 0

 .183289766134963

 1.118302416931E-04

 4.19363406349E-05

 4.19363406349E-05

 4.19363406349E-05

 0

 0

 6.98939010582E-05

 0

 0

 0

 0

 0

 0

 6.98939010582E-05

 6.98939010582E-05

 0

 0

 0

 0

 0

 .181863930553376

 0

 0

 0

 0

 0

 0

 0

 0

 0

 8.93244055524125E-02

 3.215119448679E-04

 4.19363406349E-05

 .000111830241693

 .00016774536254

 0

 0

 0

 .082097376183

 .082097376183

 6.2485147545976E-03

 0

 .00419363406349

 1.39787802116E-05

 0

 .000475278527196

 .0015656233837

 0

 0

 .000657002669947

 .000657002669947

 3.41082237163789E-02

 .000293554384444

 .000293554384444

 0

 0

 0

 4.9345094147116E-03

 .0049205306345

 1.39787802116E-05

 0

 0

 .0288522023568

 .0288522023568

 0

 0

 0

 2.79575604233E-05

 2.79575604233E-05

 0

 0

 1.258090219048E-04

 1.258090219048E-04

 5.59151208466E-05

 6.98939010582E-05

 2.12757034820834E-02

 0

 0

 0

 0

 0

 0

 0

 0

 0

 0

 0

 1.39787802116E-05

 0

 1.39787802116E-05

 0

 0

 0

 2.79575604233E-05

 2.79575604233E-05

 0

 0

 0

 0

 2.03531039881155E-02

 .00554957574402

 0

 1.39787802116E-05

 2.79575604233E-05

 0

 8.38726812698E-05

 5.59151208466E-05

 .000307533164656

 0

 .000670981450159

 .000615066329312

 .0110851727078

 .000726896571005

 8.38726812698E-05

 .000754854131428

 0

 1.39787802116E-05

 .000279575604233

 0

 0

 4.19363406349E-05

 0

 4.19363406349E-05

 .000880663153333

 0

 0

 .000181724142751

 .000698939010582

 0

 0

 0

 0

 0

 .000265596824021

 .000265596824021

 .000265596824021

 0

 2.6000531193656E-03

 1.8871353285716E-03

 0

 0

 1.39787802116E-05

 .00187315654836

 .000712917790794

 .000712917790794

 0

 0

 1.817241427513E-04

 1.817241427513E-04

 8.38726812698E-05

 0

 0

 9.78514614815E-05

 3.39824146944582E-02

 3.39824146944582E-02

 2.79575604233E-05

 1.39787802116E-05

 2.79575604233E-05

 0

 .0339125207934

 1.817241427513E-04

 1.817241427513E-04

 1.817241427513E-04

 9.78514614815E-05

 8.38726812698E-05

 1.39787802116E-05

 1.39787802116E-05

 1.39787802116E-05

 1.39787802116E-05

 0

 0

 0

 0

 0

 0

 0

 0

 1.1183024169309E-03

 2.79575604233E-05

 0

 0

 2.79575604233E-05

 2.79575604233E-05

 0

 0

 1.0903448565076E-03

 0

 0

 .000209681703175

 .000209681703175

 0

 8.806631533326E-04

 1.39787802116E-05

 .000293554384444

 .000573129988677

 0

 0

 6.6399206005205E-03

 .002893607503804

 .002893607503804

 .000293554384444

 .000293554384444

 .00260005311936

 .00260005311936

 0

 0

 3.7463130967165E-03

 3.7463130967165E-03

 3.7463130967165E-03

 1.39787802116E-05

 .00171938996603

 4.19363406349E-05

 .00197100800984

 0

 0

 0

 0

 0

 0

 0

 0

 0

 0

 4.16148286900383E-02

 4.16148286900383E-02

 0

 0

 0

 0

 0

 0

 0

 0

 0

 0

 0

 1.20357297622229E-02

 3.215119448674E-04

 9.78514614815E-05

 .000181724142751

 4.19363406349E-05

 1.17142178173555E-02

 0

 0

 .000265596824021

 9.78514614815E-05

 .00273984092148

 0

 0

 0

 .000111830241693

 .00176132630667

 .00673777206201

 0

 0

 1.118302416931E-04

 9.78514614815E-05

 9.78514614815E-05

 1.39787802116E-05

 1.39787802116E-05

 0

 0

 0

 0

 0

 0

 0

 0

 1.537665823281E-04

 0

 0

 9.78514614815E-05

 5.59151208466E-05

 4.19363406349E-05

 5.59151208466E-05

 2.79575604233E-05

 0

 0

 2.79575604233E-05

 2.93135021037942E-02

 0

 0

 0

 2.89081174776566E-02

 5.59151208466E-05

 .0258327858311

 .00301941652571

 0

 0

 4.053846261376E-04

 1.39787802116E-05

 0

 0

 .000391405845926

 0

 0

 0

 0

 2.79575604233E-05

 2.79575604233E-05

 0

 0

 0

 0

 2.79575604233E-05

 2.79575604233E-05

 2.79575604233E-05

 0

 0

 0

 0

 0

 0

 0

 0

 0

 0

 0

 0

 0

 0

 0

 0

 0

 0

 0

 6.98939010582E-05

 6.98939010582E-05

 6.98939010582E-05

 4.19363406349E-05

 4.19363406349E-05

 2.79575604233E-05

 2.79575604233E-05

 0

 0

 0

 0

 0

 0

 0

 0

 0

 0

 0

 0

 0

 0

 0

 0

 0

 5.59151208466E-05

 5.59151208466E-05

 5.59151208466E-05

 5.59151208466E-05

 5.59151208466E-05

 4.19363406349E-05

 4.19363406349E-05

 4.19363406349E-05

 4.19363406349E-05

 4.19363406349E-05

 0

 0

 0

 0

 0

 0

 0

 0

 0

 0

 0

 0

 .000265596824021

 .000265596824021

 .000265596824021

 .000265596824021

 .000265596824021

 0

 0

 0

 0

 1.7054111858189E-03

 .001663474845184

 .000922599493968

 0

 0

 0

 0

 0

 .000433342186561

 .000433342186561

 0

 0

 0

 0

 0

 0

 0

 0

 .000489257307407

 0

 .000489257307407

 .000740875351216

 0

 0

 0

 0

 5.59151208466E-05

 0

 0

 5.59151208466E-05

 8.38726812698E-05

 0

 1.39787802116E-05

 6.98939010582E-05

 5.59151208466E-05

 5.59151208466E-05

 .000181724142751

 .000181724142751

 .000363448285502

 .000139787802116

 .000223660483386

 4.19363406349E-05

 4.19363406349E-05

 4.19363406349E-05

 4.19363406349E-05

 0

 0

 0

 0

 0

 0

 0

 0

 0

 0

 0

 0

 0

 0

 0

 0

 0

 0

 0

 0

 0

 0

 0

 0

 0

 0

 0

 0

 0

 0

 0

 0

 0

 2.79575604233E-05

 2.79575604233E-05

 2.79575604233E-05

 2.79575604233E-05

 0

 2.79575604233E-05

 6.0667906118514E-03

 1.537665823279E-04

 0

 0

 0

 1.537665823279E-04

 .000111830241693

 .000111830241693

 4.19363406349E-05

 4.19363406349E-05

 5.9130240295235E-03

 5.9130240295235E-03

 .000139787802116

 .000139787802116

 .001733368746241

 .00155164460349

 .000181724142751

 4.19363406349E-05

 4.19363406349E-05

 .000643023889735

 .000643023889735

 2.79575604233E-05

 2.79575604233E-05

 .00170541118582

 .00170541118582

 .00159358094413

 .00159358094413

 2.79575604233E-05

 2.79575604233E-05

 0

 0

 0

 0

 4.3893369864544E-03

 0

 0

 0

 0

 0

 3.914058459257E-04

 0

 0

 0

 3.914058459257E-04

 1.537665823281E-04

 5.59151208466E-05

 9.78514614815E-05

 2.376392635976E-04

 1.39787802116E-05

 0

 .000223660483386

 3.9979311405287E-03

 6.98939010582E-05

 6.98939010582E-05

 6.98939010582E-05

 0

 3.3828648112165E-03

 3.3828648112165E-03

 .0006010875491

 2.79575604233E-05

 0

 1.39787802116E-05

 .00272586214127

 1.39787802116E-05

 0

 0

 0

 0

 0

 .000545172428254

 .000545172428254

 .000545172428254

 0

 0

 0

 0

 .666410389029418

 .308945021457395

 1.3279841201048E-03

 1.3279841201048E-03

 .00016774536254

 .000139787802116

 .000377427065714

 1.39787802116E-05

 0

 0

 .000111830241693

 1.39787802116E-05

 1.39787802116E-05

 .000489257307407

 .001761326306669

 .000251618043809

 .000251618043809

 .00150970826286

 .00150970826286

 .003410822371642

 .003410822371642

 .000587108768889

 .00152368704307

 .000111830241693

 0

 .000363448285503

 .000824748032487

 2.79575604233E-05

 2.79575604233E-05

 2.79575604233E-05

 2.79575604233E-05

 2.79575604233E-05

 2.79575604233E-05

 0

 0

 0

 .000195702922963

 .000195702922963

 .000195702922963

 0

 0

 .015949788221456

 .015949788221456

 .0156422550568

 .000307533164656

 6.98939010582E-05

 6.98939010582E-05

 0

 0

 0

 6.98939010582E-05

 0

 0

 0

 .00068496023037

 .00068496023037

 .00068496023037

 .187287697275495

 6.24991263261454E-02

 5.59151208466E-05

 .0434180913373

 .000824748032487

 8.38726812698E-05

 6.98939010582E-05

 .00016774536254

 6.98939010582E-05

 5.59151208466E-05

 .000587108768889

 .00606679061185

 .011099151488

 0

 0

 .012860477794685

 .0111970029495

 0

 .000894641933545

 .00076883291164

 0

 0

 0

 0

 0

 0

 .00705928400688

 .00705928400688

 4.19363406349E-05

 2.79575604233E-05

 1.39787802116E-05

 6.36314075234259E-02

 .000265596824021

 .0596334763829

 0

 0

 0

 .00369039797587

 2.79575604233E-05

 1.39787802116E-05

 0

 0

 0

 0

 5.59151208466E-05

 5.59151208466E-05

 2.79575604233E-05

 2.79575604233E-05

 4.11115926024535E-02

 .0128045626739

 .0229531571075

 0

 .000740875351217

 0

 1.39787802116E-05

 4.19363406349E-05

 0

 0

 .00455708234899

 0

 0

 0

 0

 0

 0

 0

 0

 0

 0

 2.6979045808495E-03

 2.5301592183098E-03

 8.38726812698E-05

 .00244628653704

 8.38726812698E-05

 8.38726812698E-05

 8.38726812699E-05

 2.79575604233E-05

 5.59151208466E-05

 2.1527321525976E-03

 0

 0

 0

 0

 1.39787802116E-05

 1.39787802116E-05

 .002138753372386

 .00016774536254

 .00149572948265

 .000475278527196

 9.33502942533437E-02

 9.33502942533437E-02

 .000195702922963

 0

 .000153766582328

 1.39787802116E-05

 0

 .000587108768889

 0

 5.59151208466E-05

 9.78514614815E-05

 .0916169255071

 .000363448285503

 0

 .000265596824021

 0

 0

 0

 4.1237401624373E-03

 0

 0

 0

 .00294952262466

 0

 0

 0

 .00294952262466

 .00294952262466

 0

 0

 0

 0

 0

 0

 1.817241427512E-04

 0

 0

 4.19363406349E-05

 4.19363406349E-05

 4.19363406349E-05

 4.19363406349E-05

 0

 8.38726812698E-05

 8.38726812698E-05

 1.39787802116E-05

 0

 1.39787802116E-05

 0

 0

 0

 0

 0

 0

 0

 0

 0

 0

 0

 9.924933950261E-04

 9.924933950261E-04

 1.39787802116E-05

 .000880663153333

 9.78514614815E-05

 0

 0

 0

 0

 0

 0

 0

 0

 .353341627409586

 2.79575604233E-05

 2.79575604233E-05

 2.79575604233E-05

 0

 0

 0

 0

 0

 0

 5.59151208466E-05

 0

 0

 5.59151208466E-05

 5.59151208466E-05

 .153514964284152

 .152592364790184

 .00652809035884

 0

 0

 .000587108768889

 2.79575604233E-05

 .120147615919

 .000111830241693

 1.39787802116E-05

 0

 0

 .000279575604233

 0

 2.79575604233E-05

 0

 0

 6.98939010582E-05

 0

 0

 0

 1.39787802116E-05

 0

 0

 .0213036610425

 0

 2.79575604233E-05

 .000307533164656

 .000251618043809

 0

 .00170541118582

 6.98939010582E-05

 4.19363406349E-05

 0

 .0010763660763

 .000782811691852

 .000587108768889

 .000195702922963

 0

 0

 0

 .000139787802116

 0

 .000139787802116

 0

 0

 0

 0

 0

 0

 0

 0

 0

 0

 0

 0

 0

 0

 0

 2.79575604233E-05

 2.79575604233E-05

 0

 2.79575604233E-05

 8.387268126983E-04

 8.387268126983E-04

 .000265596824021

 0

 0

 0

 .000335490725079

 0

 0

 0

 0

 .000209681703175

 2.79575604233E-05

 .000209681703175

 .000209681703175

 .000209681703175

 0

 0

 1.39787802116E-05

 1.39787802116E-05

 1.39787802116E-05

 .000615066329312

 0

 0

 .000615066329312

 .000615066329312

 0

 0

 0

 1.16583026965048E-02

 7.2130505892048E-03

 2.79575604233E-05

 .0070872415673

 0

 6.98939010582E-05

 2.79575604233E-05

 .0044452521073

 .0044452521073

 0

 0

 0

 3.5645889539726E-03

 3.5645889539726E-03

 .000265596824021

 1.39787802116E-05

 .00328501334974

 1.39787802116E-05

 1.39787802116E-05

 1.39787802116E-05

 0

 0

 0

 4.19363406349E-05

 4.19363406349E-05

 4.19363406349E-05

 1.39787802116E-05

 0

 0

 1.39787802116E-05

 1.39787802116E-05

 .182744593706808

 .165243160881833

 .156073081063

 0

 .000545172428254

 .00803779862169

 .000587108768889

 0

 1.75014328249748E-02

 8.38726812698E-05

 0

 0

 .000125809021905

 .0172917511218

 0

 0

 0

 0

 0

 0

 0

 0

 0

 0

 0

 6.8496023037073E-03

 6.8496023037073E-03

 2.3484350755546E-03

 7.548541314286E-04

 .000740875351217

 1.39787802116E-05

 .000643023889735

 .000643023889735

 .000950557054391

 .000950557054391

 .000307533164656

 .000307533164656

 .000307533164656

 1.957029229632E-04

 1.957029229632E-04

 .000125809021905

 5.59151208466E-05

 0

 1.39787802116E-05

 0

 1.5516446034919E-03

 1.5516446034919E-03

 .000950557054391

 .000125809021905

 0

 .000433342186561

 4.19363406349E-05

 .00243230775683

 .00243230775683

 .00243230775683

 0

 0

 0

 0

 1.39787802116E-05

 1.39787802116E-05

 1.39787802116E-05

 .000139787802116

 .000139787802116

 .000139787802116

 .000139787802116

 .000139787802116

 0

 0

 0

 0
