# Supplementary material for: The microbiome profiling of fungivorous black tinder fungus beetle Bolitophagus reticulatus reveals the insight into bacterial communities associated with larvae and adults
Source: PeerJ. 2019 May 7;7:e6852. doi: 10.7717/peerj.6852 (PMC6510215; doi:10.7717/peerj.6852)
Supplement: Data S1 — The first level represents the kingdom, the second level represents all phyla present in a particular sample; subsequent next levels represent the class, order, family and genus. [file peerj-07-6852-s003.zip › Supplemental_Data_S1/Im-Betula-2.html]

Javascript must be enabled to view this page.

magnitude

 1.0000000000005

 0

 0

 0

 0

 0

 0

 1.0000000000005

 1.66961632217E-05

 1.66961632217E-05

 1.66961632217E-05

 1.66961632217E-05

 1.66961632217E-05

 6.40130897919922E-02

 6.39630013023271E-02

 5.88706715197101E-02

 5.88706715197101E-02

 .00141917387384

 5.00884896651E-05

 .0217884930043

 0

 .000150265468995

 .000450796406986

 .00671185761512

 .000133569305774

 .00941663605703

 .018749791298

 0

 .005092329782617

 .005092329782617

 .0049253681504

 .000166961632217

 3.33923264434E-05

 0

 0

 0

 0

 0

 3.33923264434E-05

 3.33923264434E-05

 0

 3.33923264434E-05

 0

 0

 0

 0

 0

 0

 1.66961632217E-05

 1.66961632217E-05

 1.66961632217E-05

 1.66961632217E-05

 0

 0

 0

 0

 0

 0

 0

 0

 0

 0

 0

 .182288710054417

 1.502654689953E-04

 0

 0

 0

 0

 0

 1.168731425519E-04

 3.33923264434E-05

 3.33923264434E-05

 8.34808161085E-05

 1.66961632217E-05

 0

 6.67846528868E-05

 0

 0

 3.33923264434E-05

 0

 0

 3.33923264434E-05

 3.33923264434E-05

 .176662103048711

 0

 0

 0

 0

 0

 0

 .000116873142552

 .000116873142552

 .000116873142552

 5.98390489865454E-02

 1.66961632217E-05

 0

 0

 1.66961632217E-05

 0

 0

 0

 .0468661301633

 .0468661301633

 1.03516211974437E-02

 0

 .00434100243764

 1.66961632217E-05

 .000116873142552

 .00240424750392

 .00347280195011

 0

 0

 .00260460146258

 .00260460146258

 .026196280094847

 0

 0

 0

 0

 0

 3.6898520719951E-03

 .00363976358233

 5.00884896651E-05

 0

 0

 .0223895548803

 .0223895548803

 1.66961632217E-05

 1.66961632217E-05

 0

 1.001769793302E-04

 3.33923264434E-05

 0

 6.67846528868E-05

 0

 0

 0

 0

 5.30604067185276E-02

 0

 0

 0

 0

 0

 0

 5.00884896651E-05

 0

 5.00884896651E-05

 0

 0

 1.502654689951E-04

 0

 5.00884896651E-05

 .00010017697933

 0

 0

 6.67846528868E-05

 5.00884896651E-05

 0

 0

 1.66961632217E-05

 0

 5.27264834540938E-02

 .00429091394797

 0

 1.66961632217E-05

 8.34808161085E-05

 0

 6.67846528868E-05

 .000233746285104

 8.34808161085E-05

 3.33923264434E-05

 1.66961632217E-05

 .000217050121882

 .0429592279694

 .00322235950179

 6.67846528868E-05

 8.34808161085E-05

 0

 3.33923264434E-05

 .00123551607841

 1.66961632217E-05

 1.66961632217E-05

 0

 5.00884896651E-05

 0

 6.67846528868E-05

 0

 0

 6.67846528868E-05

 0

 0

 0

 0

 0

 0

 .000116873142552

 .000116873142552

 0

 .000116873142552

 1.54272548168234E-02

 1.53103816742714E-02

 3.33923264434E-05

 .000768023508198

 .00010017697933

 .0144087888603

 .000116873142552

 .000116873142552

 0

 0

 1.2021237519634E-03

 1.2021237519634E-03

 3.33923264434E-05

 0

 0

 .00116873142552

 .0207032423949

 .0207032423949

 0

 0

 0

 0

 .0207032423949

 0

 0

 0

 0

 0

 0

 0

 0

 0

 0

 0

 0

 0

 1.66961632217E-05

 1.66961632217E-05

 1.66961632217E-05

 1.66961632217E-05

 5.4596453734897E-03

 3.33923264434E-05

 0

 0

 3.33923264434E-05

 0

 1.66961632217E-05

 1.66961632217E-05

 5.4262530470463E-03

 0

 0

 6.678465288675E-04

 .000584365712759

 8.34808161085E-05

 4.7584065181788E-03

 3.33923264434E-05

 .000116873142552

 .00317227101212

 3.33923264434E-05

 .00140247771062

 2.5044244832585E-03

 7.513273449764E-04

 7.513273449764E-04

 .000384011754099

 .000384011754099

 .000333923264434

 .000333923264434

 3.33923264434E-05

 3.33923264434E-05

 1.7530971382821E-03

 1.7530971382821E-03

 1.7530971382821E-03

 .00010017697933

 .00143587003707

 5.00884896651E-05

 .000166961632217

 0

 0

 0

 0

 0

 0

 0

 0

 0

 0

 .182338798544081

 .182338798544081

 0

 0

 0

 0

 0

 0

 0

 0

 0

 0

 0

 2.06197615787804E-02

 1.2355160784019E-03

 5.00884896651E-05

 .00111864293585

 6.67846528868E-05

 1.93842455003785E-02

 8.34808161085E-05

 .000317227101212

 .00101846595652

 0

 .00390690219388

 0

 .00010017697933

 0

 .000667846528868

 .00497545664006

 .0083146892844

 0

 0

 .001202123751962

 0

 0

 .000651150365646

 .000651150365646

 0

 0

 0

 0

 .000550973386316

 0

 .000550973386316

 0

 5.7267839850419E-03

 3.33923264434E-05

 3.33923264434E-05

 .0023875513407

 .00218719738204

 .00020035395866

 3.3058403178985E-03

 .00322235950179

 0

 0

 8.34808161085E-05

 .154790129228297

 8.34808161085E-05

 5.00884896651E-05

 3.33923264434E-05

 .15200186997027

 .00106855444619

 .0606738571476

 .0862690753665

 .00270477844191

 .00128560456807

 2.7047784419185E-03

 1.66961632217E-05

 0

 6.67846528868E-05

 .00262129762581

 0

 0

 0

 0

 0

 0

 0

 0

 0

 0

 0

 0

 0

 0

 0

 0

 0

 0

 0

 0

 0

 0

 0

 0

 0

 0

 0

 0

 0

 0

 0

 0

 1.502654689953E-04

 1.168731425519E-04

 3.33923264434E-05

 3.33923264434E-05

 3.33923264434E-05

 0

 0

 8.34808161085E-05

 8.34808161085E-05

 8.34808161085E-05

 0

 0

 0

 0

 0

 0

 0

 3.33923264434E-05

 1.66961632217E-05

 1.66961632217E-05

 1.66961632217E-05

 1.66961632217E-05

 1.66961632217E-05

 1.66961632217E-05

 5.00884896651E-05

 5.00884896651E-05

 5.00884896651E-05

 5.00884896651E-05

 5.00884896651E-05

 .000400707917321

 .000400707917321

 .000400707917321

 0

 0

 .000400707917321

 .000400707917321

 0

 0

 0

 0

 0

 1.66961632217E-05

 1.66961632217E-05

 1.66961632217E-05

 1.66961632217E-05

 1.66961632217E-05

 5.342772230938E-04

 .000467492570207

 .000467492570207

 .000467492570207

 .000467492570207

 6.67846528868E-05

 6.67846528868E-05

 6.67846528868E-05

 6.67846528868E-05

 4.174040805429E-04

 4.174040805429E-04

 2.838347747693E-04

 0

 0

 0

 0

 0

 .000133569305774

 .000133569305774

 8.34808161085E-05

 1.66961632217E-05

 6.67846528868E-05

 0

 0

 0

 0

 0

 6.67846528868E-05

 0

 6.67846528868E-05

 1.335693057736E-04

 0

 0

 0

 0

 1.66961632217E-05

 0

 0

 1.66961632217E-05

 0

 0

 0

 0

 5.00884896651E-05

 5.00884896651E-05

 3.33923264434E-05

 3.33923264434E-05

 3.33923264434E-05

 1.66961632217E-05

 1.66961632217E-05

 0

 0

 0

 0

 0

 0

 0

 0

 0

 0

 0

 0

 0

 0

 0

 0

 0

 0

 0

 0

 0

 0

 0

 0

 0

 0

 0

 0

 0

 0

 0

 0

 0

 0

 0

 0

 0

 1.168731425519E-04

 1.168731425519E-04

 1.168731425519E-04

 1.168731425519E-04

 3.33923264434E-05

 8.34808161085E-05

 1.46592313086381E-02

 0

 0

 0

 0

 0

 0

 0

 0

 0

 1.46592313086381E-02

 1.46592313086381E-02

 .000350619427656

 .000350619427656

 .01088589842054

 .00916619360871

 .00171970481183

 1.66961632217E-05

 1.66961632217E-05

 .000500884896651

 .000500884896651

 3.33923264434E-05

 3.33923264434E-05

 .00212041272915

 .00212041272915

 .000651150365646

 .000651150365646

 .00010017697933

 .00010017697933

 0

 0

 0

 0

 3.0887901960145E-03

 5.00884896651E-05

 5.00884896651E-05

 5.00884896651E-05

 5.00884896651E-05

 0

 3.506194276561E-04

 0

 0

 0

 3.506194276561E-04

 1.502654689957E-04

 .000133569305774

 1.66961632217E-05

 2.003539586604E-04

 6.67846528868E-05

 5.00884896651E-05

 8.34808161085E-05

 2.6880822786933E-03

 3.33923264434E-05

 3.33923264434E-05

 3.33923264434E-05

 0

 1.6362239957264E-03

 1.6362239957264E-03

 .000350619427656

 .000183657795439

 0

 .000116873142552

 .000951681303636

 3.33923264434E-05

 0

 0

 0

 0

 0

 1.0184659565235E-03

 .00078471967142

 .00078471967142

 2.337462851035E-04

 .000150265468995

 6.67846528868E-05

 1.66961632217E-05

 .529101412495902

 .235833305506398

 8.348081610841E-04

 8.348081610841E-04

 6.67846528868E-05

 5.00884896651E-05

 5.00884896651E-05

 0

 1.66961632217E-05

 1.66961632217E-05

 1.66961632217E-05

 .000417404080542

 0

 .00020035395866

 .00218719738204

 0

 0

 .00218719738204

 .00218719738204

 .005993922596585

 .005993922596585

 .00155274317962

 .00098507363008

 .000317227101212

 .000584365712759

 .000734631181754

 .00181988179116

 1.66961632217E-05

 1.66961632217E-05

 1.66961632217E-05

 0

 0

 0

 0

 0

 0

 .000651150365646

 .000651150365646

 .000651150365646

 0

 0

 .01642902461016

 .01642902461016

 .0162286706515

 .00020035395866

 2.003539586604E-04

 2.003539586604E-04

 0

 0

 3.33923264434E-05

 .000166961632217

 0

 0

 0

 .000350619427656

 .000350619427656

 .000350619427656

 .100444117941641

 2.85504391090874E-02

 0

 .017247136608

 .000434100243764

 .00020035395866

 .00010017697933

 .00030053093799

 .000183657795439

 3.33923264434E-05

 .000601061875981

 .00345610578689

 .00599392259659

 0

 0

 1.00510902594591E-02

 .00763014659231

 5.00884896651E-05

 .00183657795439

 .000534277223094

 1.66961632217E-05

 1.66961632217E-05

 0

 0

 0

 0

 .00290513240057

 .00290513240057

 5.00884896651E-05

 3.33923264434E-05

 1.66961632217E-05

 2.73316191938586E-02

 .000150265468995

 .0247437138945

 0

 3.33923264434E-05

 0

 .00230407052459

 0

 3.33923264434E-05

 0

 1.66961632217E-05

 0

 5.00884896651E-05

 0

 0

 0

 0

 3.15390523257792E-02

 .018749791298

 .0100343940962

 0

 .000584365712759

 1.66961632217E-05

 1.66961632217E-05

 6.67846528868E-05

 0

 0

 .00207032423949

 0

 0

 0

 0

 0

 0

 0

 0

 0

 0

 2.7381707683627E-03

 .000801415834641

 0

 .000801415834641

 1.66961632217E-05

 1.66961632217E-05

 .0019200587705

 .00113533909908

 .00078471967142

 8.3981701005053E-03

 6.67846528868E-05

 6.67846528868E-05

 1.66961632217E-05

 1.66961632217E-05

 1.66961632217E-05

 1.66961632217E-05

 8.2979931211751E-03

 5.00884896651E-05

 .00646141516679

 .00178648946472

 9.75890740308357E-02

 9.75890740308357E-02

 .000133569305774

 0

 .000333923264434

 8.34808161085E-05

 0

 .000150265468995

 3.33923264434E-05

 1.66961632217E-05

 5.00884896651E-05

 .0962533809731

 .000384011754099

 0

 .000150265468995

 0

 0

 0

 7.2962233278884E-03

 0

 0

 0

 1.1520352623017E-03

 1.66961632217E-05

 1.66961632217E-05

 0

 .00113533909908

 .00113533909908

 0

 0

 0

 0

 0

 0

 6.177580392033E-04

 5.00884896651E-05

 5.00884896651E-05

 .000333923264434

 .000333923264434

 3.33923264434E-05

 0

 3.33923264434E-05

 0

 0

 1.836577954391E-04

 5.00884896651E-05

 .000133569305774

 0

 0

 0

 0

 0

 0

 1.66961632217E-05

 1.66961632217E-05

 0

 0

 0

 5.5264300263834E-03

 5.5097338631617E-03

 .00293852472702

 .00255451297292

 1.66961632217E-05

 1.66961632217E-05

 1.66961632217E-05

 0

 0

 0

 0

 0

 0

 .285971883661615

 0

 0

 0

 0

 0

 0

 0

 0

 0

 1.66961632217E-05

 0

 0

 1.66961632217E-05

 1.66961632217E-05

 .127091194443981

 .126406651751892

 .0102347480549

 0

 3.33923264434E-05

 .000333923264434

 5.00884896651E-05

 .102163822754

 3.33923264434E-05

 .000217050121882

 0

 5.00884896651E-05

 .000768023508198

 3.33923264434E-05

 0

 0

 6.67846528868E-05

 .00278825925802

 1.66961632217E-05

 6.67846528868E-05

 0

 .000717935018533

 0

 5.00884896651E-05

 .00437439476408

 0

 8.34808161085E-05

 .000166961632217

 .000968377466858

 0

 .00190336260727

 .000617758039203

 0

 1.66961632217E-05

 .000651150365646

 .000450796406985

 .000250442448325

 .00020035395866

 1.66961632217E-05

 0

 1.66961632217E-05

 .000217050121882

 0

 .000217050121882

 0

 0

 0

 0

 0

 0

 0

 0

 0

 .00020035395866

 .00020035395866

 .00020035395866

 0

 0

 0

 6.67846528868E-05

 6.67846528868E-05

 3.33923264434E-05

 3.33923264434E-05

 3.7399405616545E-03

 3.7399405616545E-03

 .000550973386316

 0

 0

 1.66961632217E-05

 3.33923264434E-05

 0

 0

 1.66961632217E-05

 0

 .00310548635923

 1.66961632217E-05

 1.66961632217E-05

 1.66961632217E-05

 1.66961632217E-05

 0

 0

 .000116873142552

 .000116873142552

 .000116873142552

 .00030053093799

 0

 0

 .00030053093799

 .00030053093799

 0

 0

 0

 5.58486659765217E-02

 1.37242461682217E-02

 0

 .013707550005

 0

 0

 1.66961632217E-05

 .0421244198083

 .0421244198083

 0

 0

 0

 4.174040805419E-04

 4.174040805419E-04

 6.67846528868E-05

 5.00884896651E-05

 .00030053093799

 0

 0

 0

 0

 0

 0

 3.33923264434E-05

 3.33923264434E-05

 3.33923264434E-05

 1.66961632217E-05

 1.66961632217E-05

 1.66961632217E-05

 0

 0

 9.81066550907195E-02

 8.97752696430927E-02

 .06431362073

 0

 .000701238855311

 .0150766353892

 .00966707850536

 1.66961632217E-05

 8.3313854476268E-03

 6.67846528868E-05

 0

 0

 .00345610578689

 .00480849500785

 0

 0

 0

 0

 0

 0

 0

 0

 0

 0

 0

 2.01188766821388E-02

 2.01188766821388E-02

 5.1591144355054E-03

 5.509733863154E-04

 .000517581059872

 3.33923264434E-05

 .00263799378903

 .00263799378903

 .00197014726016

 .00197014726016

 .000634454202424

 .000634454202424

 .000634454202424

 4.6415333756289E-03

 4.6415333756289E-03

 .00435769860086

 .000166961632217

 5.00884896651E-05

 6.67846528868E-05

 0

 .000567669549538

 .000567669549538

 .000283834774769

 0

 0

 .000283834774769

 0

 .009032624302934

 .0083146892844

 .0083146892844

 .000717935018534

 .000450796406986

 .000133569305774

 .000133569305774

 8.34808161085E-05

 8.34808161085E-05

 8.34808161085E-05

 1.836577954384E-04

 .000150265468995

 .000150265468995

 .000150265468995

 .000150265468995

 3.33923264434E-05

 3.33923264434E-05

 3.33923264434E-05

 3.33923264434E-05
