# Supplementary material for: The microbiome profiling of fungivorous black tinder fungus beetle Bolitophagus reticulatus reveals the insight into bacterial communities associated with larvae and adults
Source: PeerJ. 2019 May 7;7:e6852. doi: 10.7717/peerj.6852 (PMC6510215; doi:10.7717/peerj.6852)
Supplement: Data S1 — The first level represents the kingdom, the second level represents all phyla present in a particular sample; subsequent next levels represent the class, order, family and genus. [file peerj-07-6852-s003.zip › Supplemental_Data_S1/Im-Betula-3.html]

Javascript must be enabled to view this page.

magnitude

 1

 0

 0

 0

 0

 0

 0

 1

 1.35398613518E-05

 1.35398613518E-05

 1.35398613518E-05

 1.35398613518E-05

 1.35398613518E-05

 .106463929809392

 .106436850086688

 .102916486135214

 .102916486135214

 .00217991767764

 .00014893847487

 .0746452556326

 1.35398613518E-05

 9.47790294627E-05

 .000406195840555

 .00713550693241

 .000230177642981

 .0103173743501

 .00771772097054

 2.70797227036E-05

 .003520363951474

 .003520363951474

 .0033443457539

 .000176018197574

 1.35398613518E-05

 0

 0

 0

 0

 0

 1.35398613518E-05

 1.35398613518E-05

 1.35398613518E-05

 0

 0

 0

 0

 0

 0

 0

 1.35398613518E-05

 1.35398613518E-05

 1.35398613518E-05

 1.35398613518E-05

 0

 0

 0

 0

 0

 0

 0

 0

 0

 0

 0

 .169221187175034

 4.061958405542E-04

 .000270797227036

 .000270797227036

 .000270797227036

 0

 0

 1.353986135182E-04

 8.12391681109E-05

 8.12391681109E-05

 5.41594454073E-05

 5.41594454073E-05

 0

 0

 0

 0

 0

 0

 0

 0

 0

 .164076039861338

 1.35398613518E-05

 1.35398613518E-05

 1.35398613518E-05

 0

 0

 0

 8.12391681109E-05

 8.12391681109E-05

 8.12391681109E-05

 .105475519930722

 .000203097920277

 0

 4.06195840555E-05

 .000121858752166

 4.06195840555E-05

 0

 0

 .0941832755633

 .0941832755633

 9.3154246100528E-03

 0

 .00490142980936

 1.35398613518E-05

 .000230177642981

 .00127274696707

 .00289753032929

 1.35398613518E-05

 1.35398613518E-05

 .00176018197574

 .00176018197574

 2.56309575389693E-02

 .000243717504333

 .000243717504333

 5.41594454073E-05

 0

 5.41594454073E-05

 4.2650563258218E-03

 .00425151646447

 1.35398613518E-05

 0

 0

 .021013864818

 .021013864818

 4.06195840554E-05

 1.35398613518E-05

 2.70797227036E-05

 1.35398613518E-05

 1.35398613518E-05

 0

 0

 0

 0

 0

 0

 1.03579939341396E-02

 0

 0

 0

 0

 2.70797227036E-05

 2.70797227036E-05

 1.35398613518E-05

 0

 1.35398613518E-05

 0

 0

 2.301776429806E-04

 4.06195840555E-05

 .000121858752166

 6.76993067591E-05

 2.70797227036E-05

 2.70797227036E-05

 0

 0

 0

 0

 0

 0

 9.6403812824938E-03

 .0029787694974

 1.35398613518E-05

 1.35398613518E-05

 1.35398613518E-05

 1.35398613518E-05

 4.06195840555E-05

 .00014893847487

 4.06195840555E-05

 1.35398613518E-05

 .000257257365685

 .000365576256499

 .00477957105719

 .000555134315425

 1.35398613518E-05

 .000243717504333

 0

 0

 9.47790294627E-05

 0

 1.35398613518E-05

 0

 4.06195840555E-05

 0

 2.572573656842E-04

 0

 2.70797227036E-05

 0

 .000203097920277

 0

 0

 2.70797227036E-05

 .000162478336222

 .000162478336222

 8.12391681109E-05

 8.12391681109E-05

 8.12391681109E-05

 0

 6.4449740034653E-03

 6.2960355285953E-03

 .000121858752166

 .000162478336222

 5.41594454073E-05

 .0059575389948

 .00014893847487

 .00014893847487

 0

 0

 .000825931542461

 .000825931542461

 0

 0

 0

 .000825931542461

 1.51646447140072E-02

 1.51646447140072E-02

 1.35398613518E-05

 1.35398613518E-05

 2.70797227036E-05

 0

 .0151104852686

 1.35398613518E-05

 1.35398613518E-05

 1.35398613518E-05

 0

 1.35398613518E-05

 0

 0

 0

 0

 0

 0

 0

 0

 0

 0

 0

 0

 .00472541161179

 .000081239168111

 0

 0

 .000081239168111

 0

 4.06195840555E-05

 4.06195840555E-05

 .004644172443679

 0

 0

 .000257257365685

 .000257257365685

 0

 .004386915077994

 .000135398613518

 .000162478336222

 .00361514298094

 0

 .000473895147314

 3.7911611785116E-03

 .002085138648183

 .002085138648183

 .000514514731369

 .000514514731369

 .00139460571924

 .00139460571924

 .000176018197574

 .000176018197574

 1.7060225303286E-03

 1.7060225303286E-03

 1.7060225303286E-03

 .00014893847487

 .0011238084922

 2.70797227036E-05

 .000406195840555

 0

 0

 0

 0

 0

 0

 0

 0

 0

 0

 .126909120450647

 .126909120450647

 0

 0

 0

 0

 0

 0

 0

 0

 5.41594454073E-05

 5.41594454073E-05

 5.41594454073E-05

 1.50834055459335E-02

 4.061958405543E-04

 9.47790294627E-05

 .000284337088388

 2.70797227036E-05

 1.46772097053792E-02

 6.76993067591E-05

 2.70797227036E-05

 .000189558058925

 5.41594454073E-05

 .00564612218371

 0

 2.70797227036E-05

 2.70797227036E-05

 .000500974870017

 .00281629116118

 .00532116551127

 0

 0

 2.6673526863093E-03

 0

 0

 2.3559358752173E-03

 .00230177642981

 0

 5.41594454073E-05

 0

 0

 .000311416811092

 0

 .000311416811092

 0

 2.5184142114436E-03

 6.76993067591E-05

 6.76993067591E-05

 1.2862868284236E-03

 .00125920710572

 2.70797227036E-05

 1.1644280762609E-03

 .00108318890815

 0

 0

 8.12391681109E-05

 .106585788561554

 0

 0

 0

 .105678617850982

 .00044681542461

 .074198440208

 .0301261915078

 .000812391681109

 9.47790294627E-05

 9.071707105719E-04

 5.41594454073E-05

 2.70797227036E-05

 0

 .000825931542461

 0

 0

 0

 0

 0

 0

 0

 0

 0

 0

 0

 0

 0

 0

 0

 0

 0

 0

 0

 0

 0

 0

 0

 0

 0

 0

 0

 0

 0

 0

 0

 0

 2.707972270364E-04

 4.06195840555E-05

 4.06195840555E-05

 4.06195840555E-05

 4.06195840555E-05

 0

 0

 0

 0

 0

 9.47790294627E-05

 9.47790294627E-05

 9.47790294627E-05

 0

 9.47790294627E-05

 0

 0

 1.353986135182E-04

 8.12391681109E-05

 8.12391681109E-05

 8.12391681109E-05

 5.41594454073E-05

 5.41594454073E-05

 5.41594454073E-05

 0

 0

 0

 0

 0

 0

 0

 0

 0

 0

 0

 0

 0

 0

 0

 0

 0

 0

 0

 0

 0

 0

 .000758232235702

 .000582214038128

 .000582214038128

 .000582214038128

 .000582214038128

 .000176018197574

 .000176018197574

 .000176018197574

 .000176018197574

 8.123916811091E-04

 7.446923743501E-04

 6.363734835351E-04

 0

 0

 .000189558058925

 0

 .000189558058925

 5.41594454073E-05

 5.41594454073E-05

 1.624783362215E-04

 4.06195840555E-05

 .000121858752166

 5.41594454073E-05

 5.41594454073E-05

 0

 0

 0

 .000176018197574

 0

 .000176018197574

 .000108318890815

 0

 0

 0

 0

 0

 0

 0

 0

 0

 0

 0

 0

 0

 0

 0

 0

 .000108318890815

 0

 .000108318890815

 4.06195840554E-05

 4.06195840554E-05

 1.35398613518E-05

 0

 1.35398613518E-05

 0

 0

 0

 0

 0

 2.70797227036E-05

 2.70797227036E-05

 0

 0

 0

 0

 0

 0

 0

 0

 0

 0

 0

 0

 2.70797227036E-05

 2.70797227036E-05

 0

 0

 2.70797227036E-05

 2.70797227036E-05

 0

 0

 0

 0

 0

 0

 0

 1.218587521664E-04

 1.218587521664E-04

 1.218587521664E-04

 1.218587521664E-04

 8.12391681109E-05

 4.06195840555E-05

 1.42845537261748E-02

 1.353986135182E-04

 8.12391681109E-05

 8.12391681109E-05

 8.12391681109E-05

 5.41594454073E-05

 5.41594454073E-05

 5.41594454073E-05

 0

 0

 1.41491551126566E-02

 1.41491551126566E-02

 .00014893847487

 .00014893847487

 .01057463171577

 .00908524696707

 .0014893847487

 9.47790294627E-05

 9.47790294627E-05

 .000541594454073

 .000541594454073

 1.35398613518E-05

 1.35398613518E-05

 .00154354419411

 .00154354419411

 .00116442807626

 .00116442807626

 6.76993067591E-05

 6.76993067591E-05

 0

 0

 0

 0

 4.5087738301559E-03

 0

 0

 0

 0

 0

 1.1644280762565E-03

 0

 0

 0

 1.1644280762565E-03

 5.686741767767E-04

 .000473895147314

 9.47790294627E-05

 5.957538994798E-04

 .000135398613518

 1.35398613518E-05

 .00044681542461

 3.3443457538994E-03

 .000135398613518

 .000135398613518

 .000135398613518

 0

 2.4236351819755E-03

 2.4236351819755E-03

 .000568674176776

 .000176018197574

 0

 4.06195840555E-05

 .00163832322357

 0

 5.41594454073E-05

 5.41594454073E-05

 5.41594454073E-05

 0

 0

 7.311525129986E-04

 .000704072790295

 .000704072790295

 2.70797227036E-05

 0

 2.70797227036E-05

 0

 .557544410745171

 .240183600519958

 1.4216854419419E-03

 1.4216854419419E-03

 4.06195840555E-05

 .000135398613518

 .000135398613518

 1.35398613518E-05

 .000257257365685

 1.35398613518E-05

 1.35398613518E-05

 .000108318890815

 0

 .000704072790295

 .00243717504333

 0

 0

 .00243717504333

 .00243717504333

 .006106477469669

 .006106477469669

 .000663453206239

 .00181434142114

 .000257257365685

 .000189558058925

 .00105610918544

 .00212575823224

 4.06195840555E-05

 4.06195840555E-05

 4.06195840555E-05

 0

 0

 0

 0

 0

 0

 .000121858752166

 .000121858752166

 .000121858752166

 0

 0

 .018915186308444

 .018915186308444

 .018590229636

 .000324956672444

 8.12391681109E-05

 8.12391681109E-05

 0

 2.70797227036E-05

 0

 5.41594454073E-05

 0

 0

 0

 .00059575389948

 .00059575389948

 .00059575389948

 .135642331022589

 4.73082755632866E-02

 6.76993067591E-05

 .0300720320624

 .000108318890815

 .000121858752166

 .000162478336222

 .00014893847487

 2.70797227036E-05

 2.70797227036E-05

 5.41594454073E-05

 .00622833622184

 .0102902946274

 0

 0

 1.36346403812406E-02

 .0107506499133

 2.70797227036E-05

 .00190912045061

 .000947790294627

 0

 0

 0

 0

 2.70797227036E-05

 2.70797227036E-05

 .00250487435009

 .00250487435009

 0

 0

 0

 4.12559575390318E-02

 9.47790294627E-05

 .038791702773

 0

 0

 0

 .00230177642981

 1.35398613518E-05

 1.35398613518E-05

 0

 0

 0

 4.06195840555E-05

 1.35398613518E-05

 1.35398613518E-05

 4.06195840555E-05

 4.06195840555E-05

 3.08573440208291E-02

 .0158010181976

 .0126191507799

 0

 .000514514731369

 4.06195840555E-05

 4.06195840555E-05

 6.76993067591E-05

 0

 0

 .00177372183709

 0

 0

 7.446923743498E-04

 7.446923743498E-04

 1.35398613518E-05

 .00059575389948

 .000135398613518

 0

 0

 0

 6.1200173310238E-03

 5.1451473136927E-03

 9.47790294627E-05

 .00505036828423

 6.76993067591E-05

 6.76993067591E-05

 .000907170710572

 .000176018197574

 .000731152512998

 .003628682842292

 .000324956672444

 .000324956672444

 0

 0

 0

 0

 .003303726169848

 .000162478336222

 .00242363518198

 .000717612651646

 .064327881282506

 .064327881282506

 .000324956672444

 0

 6.76993067591E-05

 2.70797227036E-05

 0

 .00140814558059

 0

 5.41594454073E-05

 .000135398613518

 .0616876083189

 .00044681542461

 0

 .000176018197574

 0

 0

 0

 3.2495667244381E-03

 0

 0

 0

 .0018549610052

 0

 0

 0

 .0018549610052

 .0018549610052

 0

 0

 0

 2.70797227036E-05

 2.70797227036E-05

 2.70797227036E-05

 .000460355285962

 6.76993067591E-05

 6.76993067591E-05

 8.12391681109E-05

 8.12391681109E-05

 0

 0

 0

 0

 0

 2.707972270365E-04

 4.06195840555E-05

 .000230177642981

 0

 0

 0

 0

 0

 0

 4.06195840555E-05

 4.06195840555E-05

 0

 0

 0

 9.071707105725E-04

 .000866551126517

 .000176018197574

 .000690532928943

 0

 4.06195840555E-05

 4.06195840555E-05

 0

 0

 0

 0

 0

 0

 .314111243500775

 0

 0

 0

 0

 0

 0

 0

 0

 0

 0

 0

 0

 0

 0

 9.54154029461929E-02

 9.48196490467131E-02

 .0106694107452

 0

 2.70797227036E-05

 .000392655979203

 .000189558058925

 .0687283362218

 4.06195840555E-05

 6.76993067591E-05

 0

 9.47790294627E-05

 .000270797227036

 2.70797227036E-05

 0

 0

 0

 .000176018197574

 0

 5.41594454073E-05

 0

 8.12391681109E-05

 0

 1.35398613518E-05

 .010506932409

 0

 4.06195840555E-05

 .000216637781629

 .000609293760832

 0

 .00139460571924

 .000555134315425

 0

 6.76993067591E-05

 .00059575389948

 .000433275563258

 .000121858752166

 .000311416811092

 0

 0

 0

 8.12391681109E-05

 0

 8.12391681109E-05

 1.35398613518E-05

 1.35398613518E-05

 6.76993067591E-05

 0

 4.06195840555E-05

 2.70797227036E-05

 0

 0

 0

 .000203097920277

 .000203097920277

 .000203097920277

 0

 0

 0

 0

 0

 0

 0

 .003899480069327

 .003899480069327

 .000582214038128

 0

 0

 1.35398613518E-05

 0

 2.70797227036E-05

 0

 2.70797227036E-05

 0

 .00324956672444

 0

 1.218587521664E-04

 1.218587521664E-04

 8.12391681109E-05

 4.06195840555E-05

 0

 4.06195840555E-05

 4.06195840555E-05

 4.06195840555E-05

 4.06195840555E-05

 0

 0

 4.06195840555E-05

 4.06195840555E-05

 0

 0

 0

 3.73970970536518E-02

 1.25649913344518E-02

 0

 .0125514514731

 0

 0

 1.35398613518E-05

 .0248321057192

 .0248321057192

 0

 0

 0

 4.5764731369128E-03

 4.5764731369128E-03

 .000528054592721

 1.35398613518E-05

 .00403487868284

 0

 0

 0

 0

 0

 0

 8.12391681109E-05

 8.12391681109E-05

 8.12391681109E-05

 2.70797227036E-05

 1.35398613518E-05

 1.35398613518E-05

 1.35398613518E-05

 1.35398613518E-05

 .172308275563321

 .168435875216706

 .0845835138648

 9.47790294627E-05

 .000676993067591

 .0387646230503

 .0443024263432

 1.35398613518E-05

 .003872400346616

 0

 0

 0

 .00293814991334

 .000934250433276

 0

 0

 0

 0

 0

 0

 0

 0

 0

 0

 0

 1.50698656845692E-02

 1.50698656845692E-02

 8.9633882149025E-03

 9.884098786825E-04

 .000947790294627

 4.06195840555E-05

 .00289753032929

 .00289753032929

 .00507744800693

 .00507744800693

 .00125920710572

 .00125920710572

 .00125920710572

 9.071707105717E-04

 9.071707105717E-04

 .000541594454073

 .00014893847487

 .000121858752166

 6.76993067591E-05

 2.70797227036E-05

 1.3539861351818E-03

 1.3539861351818E-03

 .00059575389948

 2.70797227036E-05

 2.70797227036E-05

 .000676993067591

 2.70797227036E-05

 2.5590337954896E-03

 .00241009532062

 .00241009532062

 1.489384748696E-04

 .000121858752166

 0

 2.70797227036E-05

 2.70797227036E-05

 2.70797227036E-05

 2.70797227036E-05

 .000230177642981

 .000230177642981

 .000230177642981

 .000230177642981

 .000230177642981

 0

 0

 0

 0
