# Supplementary material for: The microbiome profiling of fungivorous black tinder fungus beetle Bolitophagus reticulatus reveals the insight into bacterial communities associated with larvae and adults
Source: PeerJ. 2019 May 7;7:e6852. doi: 10.7717/peerj.6852 (PMC6510215; doi:10.7717/peerj.6852)
Supplement: Data S1 — The first level represents the kingdom, the second level represents all phyla present in a particular sample; subsequent next levels represent the class, order, family and genus. [file peerj-07-6852-s003.zip › Supplemental_Data_S1/Im-Fagus-1.html]

Javascript must be enabled to view this page.

magnitude

 1.00000000000031

 0

 0

 0

 0

 0

 0

 1.00000000000031

 .0001627306198

 .0001627306198

 .0001627306198

 .0001627306198

 .0001627306198

 .001667988852953

 .001667988852953

 .001301844958402

 .001301844958402

 .000142389292325

 0

 .000793311771526

 0

 0

 .000345802567076

 0

 0

 .000020341327475

 0

 0

 .000366143894551

 .000366143894551

 .000366143894551

 0

 0

 0

 0

 0

 0

 0

 0

 0

 0

 0

 0

 0

 0

 0

 0

 0

 0

 0

 0

 0

 0

 0

 0

 0

 0

 0

 0

 0

 0

 0

 0

 .192367933931656

 .0003254612396

 .0003254612396

 .0003254612396

 .0003254612396

 0

 0

 0

 0

 0

 0

 0

 0

 0

 0

 0

 0

 0

 0

 0

 0

 .188970932243325

 0

 0

 0

 0

 0

 0

 0

 0

 0

 .14480991029504

 .000264437257175

 .000101706637375

 .000020341327475

 .000142389292325

 0

 0

 0

 .119627346881

 .119627346881

 .024918126156865

 0

 .00915359736376

 .000020341327475

 0

 .00258334858933

 .0131608388763

 0

 0

 0

 0

 2.08905433168551E-02

 0

 0

 0

 0

 0

 .00172901283538

 .00172901283538

 0

 0

 0

 .0188157279144

 .0188157279144

 4.06826549501E-05

 4.06826549501E-05

 0

 .000305119912125

 .000305119912125

 0

 0

 0

 0

 0

 0

 2.10736152641271E-02

 0

 0

 0

 0

 .000020341327475

 .000020341327475

 0

 0

 0

 0

 0

 0

 0

 0

 0

 0

 0

 0

 0

 0

 0

 0

 0

 1.69443257867021E-02

 .00734321921849

 0

 0

 .000020341327475

 0

 8.13653099001E-05

 .000671263806676

 0

 0

 0

 0

 .00711946461626

 .00126116230345

 .000020341327475

 .000020341327475

 0

 0

 .000386485222026

 0

 0

 .000020341327475

 0

 0

 .00410894814995

 .0026036899168

 .00020341327475

 0

 .00109843168365

 0

 .00020341327475

 0

 0

 0

 0

 0

 0

 0

 .001464575578202

 .000691605134151

 0

 0

 .000223754602225

 .000467850531926

 .000772970444051

 .000752629116576

 .000020341327475

 0

 0

 0

 0

 0

 0

 0

 .000732287789101

 .000732287789101

 0

 0

 0

 0

 .000732287789101

 0

 0

 0

 0

 0

 0

 0

 0

 0

 0

 0

 0

 0

 .000508533186876

 .000508533186876

 .000508533186876

 .000508533186876

 .002563007261855

 0

 0

 0

 0

 0

 0

 0

 .002563007261855

 0

 0

 0

 0

 0

 .002563007261855

 0

 0

 .00237993531458

 0

 .000183071947275

 0

 0

 0

 0

 0

 0

 0

 0

 0

 0

 0

 0

 0

 0

 0

 0

 0

 0

 0

 0

 0

 8.13653099001E-05

 8.13653099001E-05

 8.13653099001E-05

 8.13653099001E-05

 8.13653099001E-05

 .042554057077746

 .042554057077746

 0

 0

 0

 0

 0

 0

 0

 0

 0

 0

 0

 .00508533186876

 0

 0

 0

 0

 .00508533186876

 0

 .0022782286772

 0

 0

 .00012204796485

 0

 0

 0

 0

 .00140355159578

 .00128150363093

 0

 0

 0

 0

 0

 0

 0

 0

 0

 0

 0

 0

 0

 0

 0

 .000589898496776

 0

 0

 .00012204796485

 0

 .00012204796485

 .000467850531926

 .000467850531926

 0

 0

 0

 .03687882671221

 0

 0

 0

 .03687882671221

 0

 .0290677569618

 .00760765647566

 0

 .00020341327475

 0

 0

 0

 0

 0

 0

 0

 0

 0

 0

 0

 0

 0

 0

 0

 0

 0

 0

 0

 0

 0

 0

 0

 0

 0

 0

 0

 0

 0

 0

 0

 0

 0

 0

 0

 0

 0

 0

 0

 0

 0

 0

 0

 0

 0

 0

 0

 0

 0

 0

 0

 0

 0

 0

 0

 0

 0

 0

 0

 0

 0

 0

 0

 0

 0

 0

 0

 0

 0

 0

 0

 0

 0

 0

 0

 0

 0

 0

 .000020341327475

 .000020341327475

 .000020341327475

 .000020341327475

 .000020341327475

 .000223754602225

 .000223754602225

 .000223754602225

 .000223754602225

 .000223754602225

 0

 0

 0

 0

 9.4790586033551E-03

 .00909257338133

 .00648888346453

 0

 0

 .000101706637375

 0

 .000101706637375

 .00461748133683

 .00461748133683

 0

 0

 0

 .000183071947275

 0

 .000183071947275

 0

 0

 .00158662354305

 0

 .00158662354305

 .0026036899168

 0

 0

 0

 0

 0

 0

 0

 0

 0

 0

 0

 0

 .000264437257175

 .000264437257175

 .0010577490287

 .0010577490287

 .001281503630925

 .000223754602225

 .0010577490287

 4.06826549501E-05

 4.06826549501E-05

 0

 0

 0

 0

 0

 4.06826549501E-05

 4.06826549501E-05

 0

 0

 0

 0

 0

 0

 0

 0

 0

 0

 0

 0

 0

 0

 0

 .000345802567075

 .000345802567075

 .000101706637375

 .000101706637375

 .0002440959297

 0

 0

 .0002440959297

 .000101706637375

 .000101706637375

 .000101706637375

 .000101706637375

 .000101706637375

 4.06826549501E-05

 4.06826549501E-05

 4.06826549501E-05

 4.06826549501E-05

 0

 4.06826549501E-05

 2.17448790708461E-02

 0

 0

 0

 0

 0

 0

 0

 0

 0

 2.17448790708461E-02

 2.17448790708461E-02

 .00705844063384

 .00705844063384

 .01401517463033

 .0120013832103

 .00201379142003

 0

 0

 4.06826549501E-05

 4.06826549501E-05

 0

 0

 .000630581151726

 .000630581151726

 0

 0

 0

 0

 0

 0

 0

 0

 8.136530990012E-04

 0

 0

 0

 0

 0

 0

 0

 0

 0

 0

 0

 0

 0

 0

 0

 0

 0

 8.136530990012E-04

 0

 0

 0

 0

 6.10239824251E-05

 6.10239824251E-05

 0

 0

 0

 0

 6.10239824251E-05

 0

 .000488191859401

 .000488191859401

 .000488191859401

 0

 0

 2.644372571751E-04

 0

 0

 2.644372571751E-04

 .000183071947275

 8.13653099001E-05

 0

 .689123492199144

 .197554972437434

 4.3937267346102E-03

 4.3937267346102E-03

 4.06826549501E-05

 4.06826549501E-05

 .00406826549501

 0

 .000101706637375

 0

 0

 0

 0

 .000142389292325

 .00262403124428

 0

 0

 .00262403124428

 .00262403124428

 .007851752405361

 .007851752405361

 0

 0

 .00551249974573

 .000752629116576

 .000142389292325

 .00144423425073

 0

 0

 0

 0

 0

 0

 0

 0

 0

 .000345802567075

 .000183071947275

 .000183071947275

 .0001627306198

 .0001627306198

 .00345802567076

 .00345802567076

 .00345802567076

 0

 0

 0

 0

 0

 0

 0

 0

 0

 0

 0

 0

 0

 .152234494823095

 .022355118895006

 0

 .0131608388763

 .00012204796485

 .0003254612396

 0

 .00028477858465

 .00020341327475

 0

 .000386485222026

 .0026850552267

 .00518703850613

 0

 0

 .005410793108357

 .00445475071703

 .000020341327475

 .000549215841826

 .000386485222026

 .000772970444051

 .000772970444051

 0

 0

 0

 0

 .00386485222026

 .00386485222026

 0

 0

 0

 .021521124468585

 .0002440959297

 .0169443257867

 .000020341327475

 0

 0

 .00431236142471

 0

 0

 0

 0

 0

 0

 0

 0

 0

 0

 .098309635686836

 .0900307154045

 .00571591302048

 0

 .000223754602225

 0

 0

 .000183071947275

 .00012204796485

 0

 .00168833018043

 0

 .000345802567076

 6.916051341511E-04

 6.916051341511E-04

 0

 .000650922479201

 0

 0

 0

 4.06826549501E-05

 .000833994426476

 0

 0

 0

 0

 0

 .000833994426476

 0

 .000833994426476

 2.644372571751E-04

 0

 0

 0

 0

 0

 0

 2.644372571751E-04

 0

 .000223754602225

 4.06826549501E-05

 2.48571021744501E-02

 2.48571021744501E-02

 .000020341327475

 0

 0

 0

 0

 0

 0

 6.10239824251E-05

 0

 .0244909582799

 0

 0

 .00028477858465

 0

 0

 0

 1.5866235430521E-03

 0

 0

 0

 4.271678769761E-04

 .000345802567076

 .000345802567076

 0

 8.13653099001E-05

 8.13653099001E-05

 0

 0

 0

 0

 0

 0

 0

 0

 0

 0

 0

 0

 0

 0

 0

 0

 0

 0

 0

 0

 0

 0

 0

 0

 0

 0

 0

 0

 0

 0

 .001159455666076

 .001159455666076

 .0002440959297

 .000915359736376

 0

 0

 0

 0

 0

 0

 0

 0

 0

 .489981896218659

 0

 0

 0

 0

 0

 0

 0

 0

 0

 0

 0

 0

 0

 0

 .447163401883707

 .446980329936432

 .00604137426008

 0

 0

 .000183071947275

 0

 .388682085393

 8.13653099001E-05

 .000142389292325

 0

 0

 0

 0

 0

 0

 0

 .000183071947275

 0

 0

 0

 0

 0

 0

 .0148695103842

 .000142389292325

 .00126116230345

 0

 6.10239824251E-05

 .000488191859401

 .0327902198898

 .00134252761335

 0

 0

 .000711946461626

 0

 0

 0

 0

 0

 0

 .000183071947275

 0

 .000183071947275

 0

 0

 0

 0

 0

 0

 0

 0

 0

 0

 0

 0

 0

 0

 0

 0

 0

 0

 0

 .000813653099001

 .000813653099001

 .000447509204451

 0

 0

 0

 0

 0

 0

 0

 .000223754602225

 .000142389292325

 0

 .000020341327475

 .000020341327475

 0

 0

 .000020341327475

 .000223754602225

 .000223754602225

 .000223754602225

 .00370212160046

 0

 0

 .00370212160046

 .00370212160046

 0

 0

 0

 2.0137914200301E-03

 8.13653099001E-05

 0

 8.13653099001E-05

 0

 0

 0

 .00193242611013

 .00193242611013

 0

 0

 0

 0

 0

 0

 0

 0

 0

 0

 0

 0

 0

 0

 0

 0

 0

 0

 0

 0

 0

 0

 .03604483228576

 .03588210166596

 .00012204796485

 0

 .0003254612396

 .0277862533309

 .00764833913061

 0

 .0001627306198

 0

 0

 0

 0

 .0001627306198

 0

 6.10239824251E-05

 6.10239824251E-05

 6.10239824251E-05

 6.10239824251E-05

 6.10239824251E-05

 0

 0

 0

 0

 0

 .041557332031455

 .041557332031455

 .00335631903338

 0

 0

 0

 .0015459408881

 .0015459408881

 .00181037814528

 .00181037814528

 .000020341327475

 .000020341327475

 .000020341327475

 0

 0

 0

 0

 0

 0

 0

 0

 0

 0

 0

 0

 0

 0

 .0381806716706

 .0381806716706

 .0381806716706

 0

 0

 0

 0

 0

 0

 0

 0

 0

 0

 0

 0

 0

 0

 0

 0
