# Supplementary material for: The microbiome profiling of fungivorous black tinder fungus beetle Bolitophagus reticulatus reveals the insight into bacterial communities associated with larvae and adults
Source: PeerJ. 2019 May 7;7:e6852. doi: 10.7717/peerj.6852 (PMC6510215; doi:10.7717/peerj.6852)
Supplement: Data S1 — The first level represents the kingdom, the second level represents all phyla present in a particular sample; subsequent next levels represent the class, order, family and genus. [file peerj-07-6852-s003.zip › Supplemental_Data_S1/Im-Fagus-2.html]

Javascript must be enabled to view this page.

magnitude

 1.00000000000002

 .000116272309749

 .000116272309749

 .000116272309749

 .000116272309749

 .000116272309749

 .000116272309749

 .999883727690267

 .000193787182916

 .000193787182916

 .000193787182916

 .000193787182916

 .000193787182916

 1.07745673701094E-02

 1.05807801871941E-02

 1.05807801871941E-02

 1.05807801871941E-02

 .000755770013371

 0

 .00240296106815

 0

 0

 3.87574365831E-05

 .00118210181579

 0

 .00189911439257

 .00430207546073

 0

 0

 0

 0

 0

 1.937871829153E-04

 0

 0

 0

 0

 0

 1.937871829153E-04

 1.937871829153E-04

 5.81361548747E-05

 0

 .000116272309749

 0

 1.93787182916E-05

 0

 0

 0

 0

 0

 0

 0

 0

 0

 0

 0

 0

 0

 0

 0

 0

 0

 0

 .164079607774783

 .000503846675581

 .000174408464624

 .000174408464624

 .000174408464624

 0

 0

 .000329438210957

 0

 0

 .000329438210957

 0

 0

 .000329438210957

 0

 0

 0

 0

 0

 0

 0

 .158692324089722

 0

 0

 0

 0

 0

 0

 0

 0

 0

 .1035986279868

 9.68935914579E-05

 0

 9.68935914579E-05

 0

 0

 0

 0

 .086797279228

 .086797279228

 1.67044551673426E-02

 0

 .00674379396547

 1.93787182916E-05

 0

 .000387574365831

 .00955370811775

 0

 0

 0

 0

 .026665116369181

 .000135651028041

 .000135651028041

 0

 0

 0

 .00036819564754

 .00036819564754

 0

 0

 0

 .0261612696936

 .0261612696936

 0

 0

 0

 0

 0

 0

 0

 .000310059492665

 .000310059492665

 0

 .000310059492665

 .02536674224367

 0

 0

 .000174408464624

 .000174408464624

 .000174408464624

 .000174408464624

 0

 0

 0

 0

 0

 0

 0

 0

 0

 0

 0

 0

 0

 0

 0

 0

 0

 2.20917388523957E-02

 .00808092552759

 0

 1.93787182916E-05

 3.87574365831E-05

 0

 7.75148731663E-05

 .000813906168246

 0

 0

 .000174408464624

 .000329438210957

 .00792589578125

 .00416642443269

 3.87574365831E-05

 .000290680774374

 1.93787182916E-05

 0

 .000116272309749

 0

 0

 0

 0

 0

 2.9261864620263E-03

 .00174408464624

 7.75148731663E-05

 0

 .000872042323121

 0

 .000232544619499

 0

 0

 0

 0

 0

 0

 0

 1.6665697730779E-03

 9.68935914579E-05

 0

 0

 0

 9.68935914579E-05

 .00156967618162

 .00156967618162

 0

 0

 .000174408464624

 .000174408464624

 0

 0

 0

 .000174408464624

 .000910799759704

 .000910799759704

 0

 0

 0

 0

 .000910799759704

 .000445710520706

 .000445710520706

 .000445710520706

 0

 .000445710520706

 0

 0

 0

 0

 0

 0

 0

 0

 .000523225393872

 .000523225393872

 .000523225393872

 .000523225393872

 3.9145010949017E-03

 .000135651028041

 .000135651028041

 .000135651028041

 0

 0

 0

 0

 3.7788500668607E-03

 0

 0

 0

 0

 0

 3.7788500668607E-03

 .000193787182916

 0

 .00352692672907

 0

 5.81361548747E-05

 0

 0

 0

 0

 0

 0

 0

 0

 0

 0

 0

 0

 0

 0

 0

 0

 0

 0

 0

 0

 0

 0

 0

 0

 0

 0

 7.78636900955341E-02

 7.78636900955341E-02

 5.81361548747E-05

 5.81361548747E-05

 5.81361548747E-05

 0

 0

 0

 0

 0

 0

 0

 0

 1.08908396798638E-02

 .000135651028041

 0

 .000135651028041

 0

 1.07551886518228E-02

 3.87574365831E-05

 .00277115671569

 0

 0

 .000310059492665

 5.81361548747E-05

 0

 0

 0

 .00377885006686

 .00379822878515

 0

 0

 3.100594926647E-04

 0

 0

 5.81361548747E-05

 5.81361548747E-05

 0

 0

 0

 0

 .00025192333779

 0

 .00025192333779

 0

 3.294382109569E-04

 0

 0

 0

 0

 0

 3.294382109569E-04

 .000232544619499

 0

 9.68935914579E-05

 0

 .066275216557174

 0

 0

 0

 .066275216557174

 0

 .0618374900684

 .00426331802415

 0

 .000174408464624

 0

 0

 0

 0

 0

 0

 0

 0

 0

 7.75148731663E-05

 0

 0

 0

 0

 0

 0

 0

 0

 0

 0

 0

 0

 0

 0

 0

 0

 0

 0

 0

 7.75148731663E-05

 7.75148731663E-05

 7.75148731663E-05

 7.75148731663E-05

 0

 0

 0

 0

 9.68935914579E-05

 0

 0

 0

 0

 0

 0

 0

 0

 0

 9.68935914579E-05

 9.68935914579E-05

 9.68935914579E-05

 9.68935914579E-05

 0

 0

 0

 0

 0

 0

 0

 0

 0

 0

 0

 0

 0

 0

 0

 0

 0

 0

 0

 0

 0

 0

 5.81361548747E-05

 5.81361548747E-05

 5.81361548747E-05

 5.81361548747E-05

 5.81361548747E-05

 0

 0

 0

 0

 0

 .000542604112164

 .000542604112164

 .000542604112164

 .000542604112164

 .000542604112164

 0

 0

 0

 0

 1.9960079840286E-03

 1.5115400267396E-03

 .001356510280407

 0

 0

 0

 0

 0

 .000329438210957

 .000329438210957

 0

 0

 0

 0

 0

 0

 0

 0

 .00102707206945

 0

 .00102707206945

 1.550297463326E-04

 0

 0

 0

 0

 0

 0

 0

 0

 0

 0

 0

 0

 0

 0

 1.93787182916E-05

 1.93787182916E-05

 .000135651028041

 .000135651028041

 0

 .000484467957289

 .000484467957289

 0

 0

 0

 0

 0

 .00036819564754

 .00036819564754

 0

 0

 0

 0

 0

 0

 0

 0

 .000116272309749

 0

 .000116272309749

 0

 0

 0

 0

 0

 0

 0

 0

 0

 0

 0

 0

 0

 0

 0

 0

 0

 1.356510280406E-04

 1.356510280406E-04

 1.356510280406E-04

 1.356510280406E-04

 .000116272309749

 1.93787182916E-05

 1.04063717225726E-02

 1.93787182916E-05

 0

 0

 0

 1.93787182916E-05

 0

 0

 1.93787182916E-05

 1.93787182916E-05

 .010386993004281

 .010386993004281

 .00261612696936

 .00261612696936

 .00664690037401

 .00561982830456

 .00102707206945

 0

 0

 .000329438210957

 .000329438210957

 0

 0

 .000581361548747

 .000581361548747

 0

 0

 .000213165901207

 .000213165901207

 0

 0

 0

 0

 6.201189853301E-04

 0

 0

 0

 0

 0

 0

 0

 0

 0

 0

 0

 0

 0

 0

 0

 0

 0

 6.201189853301E-04

 0

 0

 0

 0

 .000213165901207

 .000213165901207

 .000213165901207

 0

 0

 0

 0

 0

 4.069530841231E-04

 4.069530841231E-04

 .000155029746333

 .000213165901207

 3.87574365831E-05

 0

 0

 0

 0

 0

 0

 0

 .70040501521227

 .21186752708173

 5.6973431777219E-03

 5.6973431777219E-03

 .000271302056082

 9.68935914579E-05

 .0050578454741

 0

 0

 0

 0

 .000116272309749

 0

 .000155029746333

 .00098831463287

 0

 0

 .00098831463287

 .00098831463287

 .0016084336182

 .0016084336182

 0

 0

 .00036819564754

 .000116272309749

 .000930178477995

 .000193787182916

 0

 0

 0

 0

 0

 0

 0

 0

 0

 .00112396566091

 0

 0

 .00112396566091

 .00112396566091

 .009747495300661

 .009747495300661

 .00935992093483

 .000387574365831

 0

 0

 0

 0

 0

 0

 0

 0

 0

 .000329438210957

 .000329438210957

 .000329438210957

 9.95872333004043E-02

 .011840396876154

 0

 .00662752165572

 0

 .00062011898533

 .000135651028041

 .000658876421913

 0

 0

 0

 .00133713156212

 .00246109722303

 0

 0

 2.3060674766927E-03

 .00189911439257

 0

 5.81361548747E-05

 .000348816929248

 1.93787182916E-05

 0

 1.93787182916E-05

 0

 0

 0

 .000891421041412

 .000891421041412

 0

 0

 0

 .007809623471503

 .000445710520706

 .0059686452338

 0

 0

 0

 .00118210181579

 0

 0

 0

 .000213165901207

 0

 0

 0

 0

 0

 0

 .076720345716351

 .0710811386935

 .0041470457144

 0

 .000561982830456

 0

 0

 .000406953084123

 0

 0

 .000523225393872

 0

 0

 6.588764219137E-04

 6.588764219137E-04

 0

 3.87574365831E-05

 .000600740267039

 0

 0

 1.93787182916E-05

 .00218979516695

 0

 0

 0

 0

 0

 .00218979516695

 0

 .00218979516695

 .001705327209659

 0

 0

 0

 0

 0

 0

 .001705327209659

 .000329438210957

 .000968935914579

 .000406953084123

 8.82313043814831E-02

 8.82313043814831E-02

 0

 0

 0

 0

 0

 0

 0

 3.87574365831E-05

 0

 .0881925469449

 0

 0

 0

 0

 0

 0

 .001530918745034

 0

 0

 0

 .000542604112164

 .000116272309749

 .000116272309749

 0

 .000426331802415

 .000426331802415

 0

 0

 0

 0

 0

 0

 0

 0

 0

 0

 0

 0

 0

 0

 0

 0

 0

 0

 0

 0

 0

 0

 0

 0

 0

 0

 0

 0

 0

 0

 .00098831463287

 .00098831463287

 .000406953084123

 .000581361548747

 0

 0

 0

 0

 0

 0

 0

 0

 0

 .487006569385506

 0

 0

 0

 .000155029746333

 .000155029746333

 .000155029746333

 0

 0

 0

 0

 0

 0

 0

 0

 .384590043214615

 .384066817820743

 .00631746216305

 0

 0

 .000290680774374

 0

 .333507741798

 5.81361548747E-05

 0

 0

 1.93787182916E-05

 1.93787182916E-05

 0

 0

 0

 0

 .000193787182916

 0

 0

 0

 0

 .000116272309749

 0

 .0113171714823

 .000174408464624

 .000465089238998

 7.75148731663E-05

 7.75148731663E-05

 0

 .0292231071837

 .00133713156212

 0

 0

 .000872042323121

 0

 0

 0

 3.87574365831E-05

 3.87574365831E-05

 0

 .000465089238998

 0

 .000465089238998

 0

 0

 1.93787182916E-05

 1.93787182916E-05

 0

 0

 0

 0

 0

 0

 0

 0

 0

 0

 0

 5.81361548747E-05

 5.81361548747E-05

 0

 5.81361548747E-05

 6.007402670388E-04

 6.007402670388E-04

 3.87574365831E-05

 0

 0

 0

 0

 .000290680774374

 0

 0

 5.81361548747E-05

 .000213165901207

 0

 .000135651028041

 .000135651028041

 5.81361548747E-05

 0

 7.75148731663E-05

 1.93787182916E-05

 1.93787182916E-05

 1.93787182916E-05

 .001104586942619

 .00025192333779

 .00025192333779

 .000852663604829

 .000852663604829

 0

 0

 0

 1.14528225102832E-02

 3.87574365832E-05

 1.93787182916E-05

 0

 1.93787182916E-05

 0

 0

 .0114140650737

 .0114140650737

 0

 0

 0

 0

 0

 0

 0

 0

 0

 0

 0

 0

 0

 0

 5.81361548747E-05

 5.81361548747E-05

 5.81361548747E-05

 0

 0

 0

 0

 0

 8.88320446485343E-02

 .085557041257257

 .000193787182916

 0

 3.87574365831E-05

 .0597639672112

 .0254636358351

 9.68935914579E-05

 3.2750033912773E-03

 7.75148731663E-05

 5.81361548747E-05

 0

 0

 .00306183749007

 7.75148731663E-05

 0

 0

 0

 0

 0

 0

 0

 0

 0

 0

 3.26337616030195E-02

 3.26337616030195E-02

 3.1199736449416E-03

 1.93787182916E-05

 1.93787182916E-05

 0

 .00145340387187

 .00145340387187

 .00164719105478

 .00164719105478

 .00133713156212

 .00133713156212

 .00133713156212

 0

 0

 0

 0

 0

 0

 0

 0

 0

 0

 0

 0

 0

 0

 .0280797628045

 .0280797628045

 .0280797628045

 0

 0

 0

 0

 9.68935914579E-05

 9.68935914579E-05

 9.68935914579E-05

 0

 0

 0

 0

 0

 0

 0

 0

 0
