# Supplementary material for: The microbiome profiling of fungivorous black tinder fungus beetle Bolitophagus reticulatus reveals the insight into bacterial communities associated with larvae and adults
Source: PeerJ. 2019 May 7;7:e6852. doi: 10.7717/peerj.6852 (PMC6510215; doi:10.7717/peerj.6852)
Supplement: Data S1 — The first level represents the kingdom, the second level represents all phyla present in a particular sample; subsequent next levels represent the class, order, family and genus. [file peerj-07-6852-s003.zip › Supplemental_Data_S1/Im-Fagus-3.html]

Javascript must be enabled to view this page.

magnitude

 1.00000000000009

 0

 0

 0

 0

 0

 0

 1.00000000000009

 .000706090026478

 .000706090026478

 .000706090026478

 .000706090026478

 .000706090026478

 2.26430233491091E-02

 2.20813608280471E-02

 2.15357458075861E-02

 2.15357458075861E-02

 .000304902511434

 0

 .0162882131108

 0

 0

 0

 6.41900024071E-05

 .000561662521062

 .00393163764744

 .000385140014443

 0

 .000545615020461

 .000545615020461

 .000545615020461

 0

 .000561662521062

 0

 0

 0

 0

 0

 .000369092513841

 .000369092513841

 .000369092513841

 0

 0

 0

 0

 .000192570007221

 .000192570007221

 .000192570007221

 0

 0

 0

 0

 0

 0

 0

 0

 0

 0

 0

 0

 0

 0

 0

 .141009387787864

 1.26935729760513E-02

 .0113295354249

 0

 0

 .0113295354249

 .0113295354249

 .000882612533098

 .000882612533098

 .000882612533098

 0

 0

 0

 0

 0

 0

 4.814250180533E-04

 4.81425018053E-05

 4.81425018053E-05

 .000433282516248

 .000433282516248

 .125090267190853

 0

 0

 0

 .000240712509027

 .000240712509027

 .000240712509027

 0

 0

 0

 .034437936291387

 .00739789777742

 0

 .00335392762577

 .00207012757763

 .00197384257402

 .00218246008184

 .00218246008184

 .00964454786167

 .00964454786167

 .014795795554811

 0

 .000545615020461

 0

 0

 .00133194254995

 .0129182379844

 .000128380004814

 .000128380004814

 .000288855010832

 .000288855010832

 .012645430474196

 .000385140014443

 .000385140014443

 0

 0

 0

 .00102704003851

 .00102704003851

 0

 0

 0

 .0108481104068

 .0108481104068

 .000385140014443

 .000385140014443

 0

 0

 0

 0

 0

 1.60475006018E-05

 1.60475006018E-05

 1.60475006018E-05

 0

 2.99125411217141E-02

 0

 0

 0

 0

 0

 0

 0

 0

 0

 0

 0

 0

 0

 0

 0

 0

 0

 9.62850036107E-05

 9.62850036107E-05

 0

 0

 0

 0

 1.85509106956634E-02

 .00712509026719

 0

 0

 .000144427505416

 0

 8.02375030089E-05

 0

 0

 0

 .000240712509027

 0

 .0080879403033

 .00205408007703

 8.02375030089E-05

 .000385140014443

 0

 0

 .000320950012036

 1.60475006018E-05

 0

 1.60475006018E-05

 0

 0

 .01126534542244

 .00569686271363

 .00110727754152

 .00027280751023

 .00309716761614

 0

 .00109123004092

 0

 0

 0

 0

 0

 0

 0

 2.24985958436768E-02

 .018246008184206

 .000112332504212

 0

 .000850517531894

 .0172831581481

 4.2525876594708E-03

 .00397978014924

 1.60475006018E-05

 .000256760009629

 9.62850036107E-05

 9.62850036107E-05

 0

 6.41900024071E-05

 3.20950012036E-05

 0

 2.52427184466396E-02

 2.52427184466396E-02

 0

 0

 .000320950012036

 3.20950012036E-05

 .0248896734334

 .000385140014443

 .000385140014443

 .000385140014443

 0

 .000385140014443

 0

 0

 0

 0

 0

 0

 0

 0

 .000304902511434

 .000304902511434

 .000304902511434

 .000304902511434

 .002535505095083

 0

 0

 0

 0

 0

 0

 0

 .002535505095083

 0

 0

 0

 0

 0

 .002535505095083

 0

 .000385140014443

 .00215036508064

 0

 0

 4.81425018053E-05

 0

 0

 0

 0

 0

 0

 0

 0

 4.81425018053E-05

 4.81425018053E-05

 4.81425018053E-05

 0

 0

 0

 4.81425018053E-05

 .000112332504212

 .000112332504212

 .000112332504212

 .000112332504212

 .000112332504212

 0

 0

 0

 0

 0

 9.9334028725039E-03

 9.9334028725039E-03

 0

 0

 0

 0

 0

 0

 0

 0

 0

 0

 0

 3.7069726390079E-03

 .000433282516248

 0

 .000433282516248

 0

 3.2736901227599E-03

 .000481425018053

 3.20950012036E-05

 4.81425018053E-05

 0

 .000160475006018

 0

 0

 0

 0

 0

 .00255155259568

 0

 0

 .000577710021665

 0

 0

 0

 0

 0

 0

 0

 0

 .000577710021665

 .000256760009629

 0

 .000320950012036

 .003835352643831

 0

 0

 .00328973762337

 0

 .00328973762337

 .000545615020461

 .000545615020461

 0

 0

 0

 .001813367568

 0

 0

 0

 .001813367568

 0

 .001813367568

 0

 0

 0

 0

 0

 0

 0

 0

 0

 0

 0

 0

 1.0751825403196E-03

 5.295675198586E-04

 .000497472518655

 .000497472518655

 0

 .000497472518655

 3.20950012036E-05

 3.20950012036E-05

 3.20950012036E-05

 0

 0

 0

 0

 0

 0

 0

 .000320950012036

 .000320950012036

 .000320950012036

 .000320950012036

 0

 0

 0

 0

 .000224665008425

 .000224665008425

 .000224665008425

 .000224665008425

 .000256760009629

 0

 0

 0

 0

 0

 0

 0

 0

 0

 .000256760009629

 .000256760009629

 0

 0

 0

 .000256760009629

 .000256760009629

 0

 0

 0

 0

 0

 0

 0

 0

 0

 0

 0

 0

 0

 0

 0

 0

 0

 0

 0

 0

 0

 0

 0

 0

 0

 0

 0

 0

 0

 0

 0

 0

 0

 0

 0

 0

 0

 0

 1.46353205488258E-02

 1.46353205488258E-02

 1.19393404477258E-02

 1.60475006018E-05

 1.60475006018E-05

 0

 0

 0

 .00600176522507

 .00600176522507

 .000304902511434

 0

 .000304902511434

 0

 0

 0

 0

 0

 .00561662521062

 0

 .00561662521062

 .0026959801011

 0

 0

 0

 0

 .000128380004814

 .000128380004814

 0

 0

 .000545615020461

 0

 0

 .000545615020461

 0

 0

 .000224665008425

 .000224665008425

 .0017973200674

 .000962850036107

 .000834470031293

 0

 0

 0

 0

 0

 0

 0

 0

 0

 0

 0

 0

 0

 0

 0

 0

 0

 0

 0

 0

 0

 0

 0

 0

 0

 0

 0

 0

 0

 0

 0

 0

 0

 0

 0

 0

 0

 .000208617507823

 .000208617507823

 .000208617507823

 .000208617507823

 .000208617507823

 0

 .03588221134561

 0

 0

 0

 0

 0

 0

 0

 0

 0

 .03588221134561

 .03588221134561

 .0250020059376

 .0250020059376

 .00593757522266

 .00471796517692

 .00121961004574

 0

 0

 0

 0

 0

 0

 .00494263018535

 .00494263018535

 0

 0

 0

 0

 0

 0

 0

 0

 .002631790098693

 0

 0

 0

 0

 0

 0

 0

 0

 0

 0

 0

 0

 0

 0

 0

 0

 0

 .002631790098693

 .000561662521062

 .000561662521062

 .000561662521062

 0

 .001315895049347

 .001315895049347

 .000128380004814

 .000256760009629

 0

 0

 .000690042525877

 .000240712509027

 6.900425258769E-04

 6.900425258769E-04

 .000609805022868

 0

 8.02375030089E-05

 6.41900024071E-05

 6.41900024071E-05

 6.41900024071E-05

 0

 0

 0

 0

 .76938136885185

 .40576105271604

 1.3961325523536E-03

 1.3961325523536E-03

 0

 0

 1.60475006018E-05

 0

 0

 0

 0

 0

 1.60475006018E-05

 .00136403755115

 .000160475006018

 0

 0

 .000160475006018

 .000160475006018

 2.8885501083189E-03

 2.8885501083189E-03

 0

 0

 .00280831260531

 0

 0

 8.02375030089E-05

 0

 0

 0

 0

 0

 0

 0

 0

 0

 0

 0

 0

 0

 0

 .00282436010591

 .00282436010591

 .00282436010591

 0

 .000930755034903

 .000930755034903

 .000930755034903

 0

 0

 0

 0

 0

 0

 .00117146754393

 .00117146754393

 .00117146754393

 .339372542726502

 .154601620797603

 .000288855010832

 .0574340046538

 .00215036508064

 .00112332504212

 9.62850036107E-05

 0

 0

 0

 0

 .0340046537752

 .0595041322314

 0

 0

 3.3699751263653E-03

 .00229479258605

 0

 .00102704003851

 4.81425018053E-05

 0

 0

 0

 0

 0

 0

 .0321270962048

 .0321270962048

 0

 0

 0

 9.93500762256468E-02

 .0017652250662

 .0917435609404

 1.60475006018E-05

 .000224665008425

 0

 .00457353767151

 .00102704003851

 0

 0

 0

 0

 0

 0

 0

 0

 0

 4.99237743720869E-02

 .0265586134959

 .0171387306427

 0

 .00157265505897

 0

 3.20950012036E-05

 4.81425018053E-05

 .000288855010832

 .000240712509027

 .00369092513841

 0

 .000353045013239

 8.986600337005E-04

 8.986600337005E-04

 0

 .000529567519859

 .000256760009629

 0

 8.02375030089E-05

 3.20950012036E-05

 0

 0

 0

 0

 0

 0

 0

 0

 0

 0

 0

 0

 0

 0

 0

 0

 0

 0

 0

 0

 5.59094920965818E-02

 5.59094920965818E-02

 1.60475006018E-05

 0

 0

 0

 0

 0

 0

 .00044933001685

 0

 .054240552034

 0

 .00120356254513

 0

 .000208617507823

 .000208617507823

 .000208617507823

 .001010992537913

 0

 0

 0

 0

 0

 0

 0

 0

 0

 0

 0

 0

 0

 0

 0

 .001010992537913

 0

 0

 .00044933001685

 .00044933001685

 0

 0

 0

 0

 0

 .00017652250662

 0

 .00017652250662

 .000240712509027

 .000240712509027

 0

 0

 0

 0

 .000144427505416

 .000144427505416

 0

 0

 0

 0

 0

 0

 0

 0

 0

 0

 0

 0

 0

 0

 0

 0

 .362609323597897

 0

 0

 0

 0

 0

 0

 0

 0

 0

 .000160475006018

 0

 0

 .000160475006018

 .000160475006018

 .146930915509523

 .146353205487859

 .00319345261975

 .00017652250662

 0

 .000336997512637

 0

 .140383535264

 0

 0

 0

 0

 0

 0

 0

 0

 0

 0

 0

 4.81425018053E-05

 0

 0

 0

 0

 .000465377517452

 0

 .000802375030089

 0

 0

 0

 .0008986600337

 0

 0

 0

 4.81425018053E-05

 0

 0

 0

 .000304902511434

 0

 .000304902511434

 .00027280751023

 .00027280751023

 0

 0

 0

 0

 0

 0

 0

 0

 0

 0

 0

 0

 0

 0

 0

 0

 8.02375030089E-05

 8.02375030089E-05

 0

 8.02375030089E-05

 .001717082564391

 .001717082564391

 .000609805022868

 .000561662521062

 0

 0

 0

 0

 0

 0

 0

 .000545615020461

 0

 .000866565032496

 .000866565032496

 0

 .000866565032496

 0

 .00226269758485

 .00226269758485

 .00226269758485

 .00922731284602

 0

 0

 .00922731284602

 .00922731284602

 0

 0

 0

 4.4130626654856E-03

 3.209500120356E-04

 3.20950012036E-05

 .000288855010832

 0

 0

 0

 .00409211265345

 .00409211265345

 0

 0

 0

 0

 0

 0

 0

 0

 .000208617507823

 .000208617507823

 .000208617507823

 0

 0

 0

 .00256760009629

 .00256760009629

 .00256760009629

 0

 0

 0

 0

 0

 .194174757281992

 .193677284763337

 .000160475006018

 .00195779507342

 .000256760009629

 .00444515766669

 .185268394448

 .00158870255958

 .000497472518655

 0

 0

 .000192570007221

 .000304902511434

 0

 0

 0

 0

 0

 0

 0

 0

 0

 0

 0

 0

 .001476370055364

 .001476370055364

 .00044933001685

 .00044933001685

 .00044933001685

 0

 0

 0

 0

 0

 .000529567519859

 .000529567519859

 .000529567519859

 0

 0

 0

 0

 0

 0

 0

 .000128380004814

 .000128380004814

 .000128380004814

 0

 0

 0

 0

 .000369092513841

 .000160475006018

 .000160475006018

 .000208617507823

 .000208617507823

 0

 0

 0

 0

 0

 0

 0

 0

 0

 0

 0

 0

 0

 0
