# Supplementary material for: The microbiome profiling of fungivorous black tinder fungus beetle Bolitophagus reticulatus reveals the insight into bacterial communities associated with larvae and adults
Source: PeerJ. 2019 May 7;7:e6852. doi: 10.7717/peerj.6852 (PMC6510215; doi:10.7717/peerj.6852)
Supplement: Data S1 — The first level represents the kingdom, the second level represents all phyla present in a particular sample; subsequent next levels represent the class, order, family and genus. [file peerj-07-6852-s003.zip › Supplemental_Data_S1/L-Betula-1.html]

Javascript must be enabled to view this page.

magnitude

 1.00000000000026

 .000598992943114

 .000598992943114

 .000598992943114

 .000598992943114

 .000598992943114

 .000598992943114

 .999401007057148

 .0106321247403

 .0106321247403

 .0106321247403

 .0106321247403

 .0106321247403

 4.6421953091323E-03

 0

 0

 0

 0

 0

 0

 0

 0

 0

 0

 0

 0

 0

 0

 0

 0

 0

 0

 4.6421953091323E-03

 0

 0

 0

 0

 0

 4.6421953091323E-03

 4.6421953091323E-03

 1.87185294723E-05

 .00131029706306

 0

 .00190929000618

 .00140388971042

 0

 0

 0

 0

 0

 0

 0

 0

 0

 0

 0

 0

 0

 0

 0

 0

 0

 0

 .21354098422025

 4.6421953091306E-03

 2.7329053029546E-03

 .00269546824401

 .00269546824401

 3.74370589446E-05

 3.74370589446E-05

 0

 0

 0

 0

 0

 0

 0

 0

 0

 .001909290006176

 .000205903824196

 .000205903824196

 .00170338618198

 .00170338618198

 .206240757726048

 0

 0

 0

 0

 0

 0

 0

 0

 0

 .054096550174959

 .03496621305425

 0

 .0133650300432

 .0163599947588

 .00524118825225

 0

 0

 .00398704677761

 .00398704677761

 .015143290343099

 0

 .000149748235779

 0

 0

 .00479194354492

 .0102015985624

 0

 0

 0

 0

 .00709432267001

 0

 0

 .00228366059562

 0

 .00228366059562

 0

 0

 0

 0

 0

 .00248956441982

 .00248956441982

 0

 0

 0

 .00232109765457

 .00232109765457

 0

 0

 0

 0

 0

 0

 9.52773150141663E-02

 0

 0

 0

 0

 .000505400295753

 .000505400295753

 0

 0

 0

 0

 0

 0

 0

 0

 0

 0

 0

 2.0403197124833E-03

 .00185313441776

 0

 1.87185294723E-05

 .000168466765251

 0

 6.07790651966631E-02

 .000131029706306

 0

 3.74370589446E-05

 .000056155588417

 0

 .000056155588417

 0

 0

 9.35926473616E-05

 0

 .00241469030193

 .051756733991

 .00488553619228

 0

 0

 0

 1.87185294723E-05

 .00129157853359

 0

 0

 3.74370589446E-05

 0

 0

 3.19525298092669E-02

 1.87185294723E-05

 3.74370589446E-05

 .00909720532355

 .0227991688973

 0

 0

 0

 0

 0

 0

 0

 0

 0

 .04778840574285

 .00368755030605

 0

 0

 0

 .00368755030605

 .0441008554368

 .0441008554368

 0

 0

 .00196544559459

 .00196544559459

 0

 0

 0

 .00196544559459

 1.87185294723E-05

 1.87185294723E-05

 0

 0

 0

 0

 1.87185294723E-05

 0

 0

 0

 0

 0

 0

 0

 0

 0

 0

 0

 0

 0

 .0017595417704

 .0017595417704

 .0017595417704

 .0017595417704

 .000898489414672

 .000898489414672

 .000786178237838

 .000786178237838

 .000112311176834

 0

 0

 .000112311176834

 0

 0

 0

 0

 0

 0

 0

 0

 0

 0

 0

 0

 0

 0

 0

 0

 0

 0

 0

 0

 0

 0

 0

 0

 0

 0

 0

 0

 0

 0

 0

 0

 0

 0

 0

 0

 0

 0

 .02877037979893

 .02710443067589

 0

 0

 0

 0

 0

 0

 0

 0

 0

 0

 0

 0

 0

 0

 0

 0

 0

 0

 0

 0

 0

 0

 0

 0

 0

 0

 0

 0

 0

 0

 0

 0

 0

 0

 0

 0

 0

 0

 0

 0

 0

 0

 0

 .01008928738558

 0

 0

 .00602736649009

 0

 .00602736649009

 .00406192089549

 .00202160118301

 .00204031971248

 0

 0

 .01701514329031

 0

 0

 0

 .01701514329031

 0

 .0130280965127

 .00398704677761

 0

 0

 0

 0

 0

 0

 0

 .00166594912304

 .00166594912304

 .00166594912304

 .00166594912304

 1.8156973588123E-03

 1.8156973588123E-03

 .00179697882934

 .00179697882934

 0

 .00179697882934

 1.87185294723E-05

 1.87185294723E-05

 1.87185294723E-05

 0

 0

 0

 0

 0

 0

 0

 0

 0

 0

 0

 0

 0

 0

 0

 0

 0

 0

 0

 0

 0

 0

 0

 0

 0

 0

 0

 0

 0

 0

 0

 0

 0

 0

 0

 0

 0

 0

 0

 0

 0

 0

 0

 0

 0

 0

 0

 0

 0

 0

 0

 0

 0

 0

 0

 0

 0

 0

 0

 0

 .00159107500515

 .00159107500515

 .00159107500515

 .00159107500515

 .00159107500515

 0

 0

 0

 0

 0

 0

 0

 0

 0

 8.72657844000596E-02

 8.19684405593906E-02

 5.88884937200106E-02

 9.35926473616E-05

 9.35926473616E-05

 .0017595417704

 0

 .0017595417704

 .019972670947

 .019972670947

 0

 0

 0

 .002658031185069

 .00250828294929

 0

 0

 .000149748235779

 .03440465717018

 .00048668176628

 .0339179754039

 .02307994683938

 0

 0

 0

 0

 .00164723059356

 0

 0

 .00164723059356

 .00215263088932

 0

 0

 .00215263088932

 0

 0

 .00293880912715

 .00293880912715

 .01634127622935

 .00578402560695

 .0105572506224

 .005297343840669

 .005297343840669

 0

 0

 0

 0

 0

 .00116054882728

 0

 .00116054882728

 0

 0

 .00398704677761

 .00398704677761

 0

 0

 0

 .000149748235779

 .000149748235779

 0

 0

 0

 0

 0

 0

 0

 0

 0

 0

 0

 0

 0

 0

 0

 0

 0

 0

 0

 0

 0

 0

 0

 0

 5.8214626658956E-03

 0

 0

 0

 0

 0

 0

 0

 0

 0

 5.8214626658956E-03

 5.8214626658956E-03

 .00327574265766

 .00327574265766

 9.35926473616E-05

 9.35926473616E-05

 0

 0

 0

 .0020964753009

 .0020964753009

 0

 0

 .000355652059974

 .000355652059974

 0

 0

 0

 0

 0

 0

 0

 0

 .00269546824402

 .00269546824402

 .00269546824402

 .00269546824402

 .00134773412201

 .00134773412201

 0

 0

 0

 0

 0

 0

 0

 0

 0

 0

 0

 0

 0

 0

 0

 0

 0

 0

 0

 0

 0

 0

 0

 0

 0

 0

 0

 0

 0

 0

 0

 0

 0

 0

 0

 0

 0

 .640211145012668

 .150833910487937

 1.36832450442723E-02

 1.36832450442723E-02

 0

 .0018905714767

 0

 0

 .00569043295959

 0

 0

 0

 1.87185294723E-05

 .00608352207851

 .00217134941879

 .00217134941879

 .00217134941879

 0

 0

 1.8156973588169E-03

 1.8156973588169E-03

 3.74370589446E-05

 0

 .0017595417704

 0

 0

 1.87185294723E-05

 0

 0

 0

 0

 0

 0

 0

 0

 0

 0

 0

 0

 0

 0

 .0026393126556

 .0026393126556

 .0026393126556

 0

 0

 0

 0

 0

 0

 0

 .000935926473616

 .000935926473616

 .000935926473616

 0

 0

 0

 .12232559010158

 8.797708851993E-04

 0

 .000861052355727

 0

 0

 0

 0

 0

 0

 0

 1.87185294723E-05

 0

 0

 0

 1.9467270651243E-03

 .00159107500515

 0

 1.87185294723E-05

 .000336933530502

 1.87185294723E-05

 0

 0

 1.87185294723E-05

 0

 0

 .00219006794826

 .00219006794826

 0

 0

 0

 8.58618946895336E-02

 3.74370589446E-05

 .0848698126275

 0

 0

 0

 .000954645003089

 0

 0

 0

 0

 0

 0

 0

 0

 0

 0

 3.14284109839906E-02

 .0150122606368

 .0047357879565

 0

 .000374370589446

 0

 3.74370589446E-05

 0

 0

 0

 .0112685547423

 0

 0

 1.87185294723E-05

 1.87185294723E-05

 0

 0

 0

 0

 0

 1.87185294723E-05

 4.1555135428546E-03

 4.1555135428546E-03

 3.74370589446E-05

 .00411807648391

 0

 0

 0

 0

 0

 1.87185294723E-05

 0

 0

 0

 0

 0

 0

 1.87185294723E-05

 0

 1.87185294723E-05

 0

 3.0698388334623E-03

 3.0698388334623E-03

 1.87185294723E-05

 0

 0

 0

 0

 0

 0

 0

 0

 .00305112030399

 0

 0

 0

 0

 0

 0

 .00423038766075

 0

 0

 0

 0

 0

 0

 0

 0

 0

 0

 0

 0

 0

 0

 0

 0

 0

 0

 0

 0

 0

 0

 0

 0

 0

 0

 0

 0

 0

 0

 0

 0

 0

 0

 0

 0

 0

 0

 0

 0

 0

 0

 0

 0

 0

 0

 0

 0

 0

 .00423038766075

 .00423038766075

 .00423038766075

 .485146846863982

 0

 0

 0

 0

 0

 0

 .00117926735676

 .00117926735676

 .00117926735676

 1.61540909346446E-02

 3.74370589446E-05

 3.74370589446E-05

 .0161166538757

 .0161166538757

 .331411564307718

 .329970237538348

 .00441757295547

 0

 .00181569735882

 .000842333826255

 .00346292795238

 .307882372761

 0

 0

 0

 0

 0

 0

 .00284521647979

 .00140388971042

 0

 .00224622353668

 0

 0

 0

 0

 0

 0

 .000636430002059

 0

 0

 0

 .00342549089344

 0

 .000954645003089

 0

 0

 0

 3.74370589446E-05

 0

 0

 0

 .00144132676937

 .00144132676937

 0

 0

 0

 0

 0

 0

 0

 0

 0

 0

 0

 0

 0

 0

 0

 0

 0

 0

 0

 0

 0

 0

 0

 .03558392452686

 .03558392452686

 .0157610018157

 0

 0

 .00219006794826

 0

 0

 0

 0

 0

 .0176328547629

 0

 .000112311176834

 .000112311176834

 .000112311176834

 0

 0

 .00376242442394

 .00376242442394

 .00376242442394

 .035190835408

 0

 0

 .035190835408

 .035190835408

 0

 0

 0

 4.23787507253316E-02

 3.19525298092316E-02

 .029406809801

 0

 9.35926473616E-05

 .00245212736087

 0

 .0104262209161

 .0104262209161

 0

 0

 0

 0

 0

 0

 0

 0

 0

 0

 0

 .00146004529884

 .00146004529884

 .00146004529884

 .0113247103308

 .0113247103308

 .0113247103308

 0

 0

 0

 0

 0

 .006588922374254

 .005278625311194

 .000056155588417

 0

 0

 .00516631413436

 .000056155588417

 0

 .00131029706306

 0

 0

 0

 0

 .00131029706306

 0

 .00241469030193

 .00241469030193

 .00241469030193

 .00241469030193

 .00241469030193

 0

 0

 0

 0

 0

 0

 0

 0

 0

 0

 0

 0

 0

 0

 0

 0

 0

 0

 0

 0

 0

 0

 0

 0

 0

 0

 0

 0

 0

 0

 0

 0

 0

 0

 0

 0

 0

 0

 0

 0

 0

 0

 0

 0

 0

 0

 0

 0

 0

 0

 0
