# Supplementary material for: The microbiome profiling of fungivorous black tinder fungus beetle Bolitophagus reticulatus reveals the insight into bacterial communities associated with larvae and adults
Source: PeerJ. 2019 May 7;7:e6852. doi: 10.7717/peerj.6852 (PMC6510215; doi:10.7717/peerj.6852)
Supplement: Data S1 — The first level represents the kingdom, the second level represents all phyla present in a particular sample; subsequent next levels represent the class, order, family and genus. [file peerj-07-6852-s003.zip › Supplemental_Data_S1/L-Betula-2.html]

Javascript must be enabled to view this page.

magnitude

 1.00000000000012

 0

 0

 0

 0

 0

 0

 1.00000000000012

 .0013093118257

 .0013093118257

 .0013093118257

 .0013093118257

 .0013093118257

 1.69424950246482E-02

 .0122027862156

 0

 0

 0

 0

 0

 0

 0

 0

 0

 0

 0

 0

 0

 .0122027862156

 .0122027862156

 .0122027862156

 0

 4.7397088090482E-03

 3.5613281659182E-03

 5.23724730282E-05

 5.23724730282E-05

 .00350895569289

 .00350895569289

 .00117838064313

 .00117838064313

 .00117838064313

 0

 0

 0

 0

 0

 0

 0

 0

 0

 0

 0

 0

 0

 0

 0

 0

 0

 0

 0

 0

 0

 0

 .204174086100288

 4.1897978422582E-03

 2.4091337592982E-03

 .00235676128627

 .00235676128627

 5.23724730282E-05

 5.23724730282E-05

 .00178066408296

 0

 0

 0

 0

 0

 0

 .00178066408296

 .00178066408296

 0

 0

 0

 0

 0

 .18429873258608

 .00115219440662

 .00115219440662

 .00115219440662

 0

 0

 0

 0

 0

 0

 2.34628679166382E-02

 .01089347438987

 0

 .00387556300409

 .00701791138578

 0

 0

 0

 2.61862365141E-05

 2.61862365141E-05

 1.25432072902541E-02

 0

 2.61862365141E-05

 0

 0

 .00586571697916

 .00665130407458

 0

 0

 0

 0

 2.1734576306682E-03

 0

 0

 0

 0

 0

 0

 0

 0

 0

 0

 0

 0

 0

 0

 0

 2.1734576306682E-03

 .00212108515764

 0

 5.23724730282E-05

 0

 0

 0

 0

 .113491149052009

 0

 0

 0

 0

 .00214727139416

 .00214727139416

 0

 0

 0

 0

 0

 0

 0

 0

 0

 0

 0

 .00437310149785

 0

 0

 0

 0

 .00437310149785

 9.39562166124974E-02

 .000288048601655

 0

 5.23724730282E-05

 .00070702838588

 0

 .00013093118257

 2.61862365141E-05

 0

 7.85587095423E-05

 .000157117419085

 0

 .0826175762019

 .00730595998743

 2.61862365141E-05

 5.23724730282E-05

 0

 0

 .00238294752278

 0

 7.85587095423E-05

 5.23724730282E-05

 0

 0

 .013014559547502

 0

 .00730595998743

 .000523724730282

 .00505394364722

 .00013093118257

 0

 0

 0

 0

 0

 0

 0

 0

 .03718445584997

 .00013093118257

 0

 0

 0

 .00013093118257

 .0370535246674

 .0370535246674

 0

 0

 0

 0

 0

 0

 0

 0

 6.8346077301741E-03

 6.8346077301741E-03

 0

 0

 2.61862365141E-05

 0

 .00680842149366

 .00418979784225

 .00418979784225

 .00418979784225

 0

 .00418979784225

 0

 0

 0

 0

 0

 0

 0

 0

 .0114957578297

 .0114957578297

 .0114957578297

 .0114957578297

 0

 0

 0

 0

 0

 0

 0

 0

 0

 0

 0

 0

 0

 0

 0

 0

 0

 0

 0

 0

 0

 0

 0

 0

 0

 0

 0

 0

 0

 0

 0

 0

 0

 0

 0

 0

 0

 0

 0

 0

 0

 0

 0

 0

 0

 0

 2.12894102859413E-02

 2.12894102859413E-02

 0

 0

 0

 0

 0

 0

 0

 0

 0

 0

 0

 5.23724730282E-05

 2.61862365141E-05

 0

 2.61862365141E-05

 0

 2.61862365141E-05

 0

 0

 0

 2.61862365141E-05

 0

 0

 0

 0

 0

 0

 0

 0

 0

 .000183303655599

 0

 0

 .000183303655599

 0

 .000183303655599

 0

 0

 0

 0

 0

 0

 0

 .0210275479208

 0

 0

 .0210275479208

 0

 .0210275479208

 0

 0

 0

 0

 0

 2.61862365141E-05

 0

 0

 0

 2.61862365141E-05

 0

 0

 2.61862365141E-05

 0

 0

 0

 0

 0

 0

 0

 0

 0

 0

 0

 .01288362836489

 .01288362836489

 .01288362836489

 .01288362836489

 .00259243741489

 .01029119095

 0

 0

 0

 0

 0

 0

 0

 0

 0

 0

 0

 0

 0

 0

 0

 0

 0

 0

 0

 0

 0

 0

 0

 0

 0

 0

 0

 0

 0

 0

 0

 0

 0

 0

 0

 0

 0

 0

 0

 0

 0

 0

 0

 0

 0

 0

 0

 0

 0

 0

 0

 0

 0

 0

 0

 0

 0

 0

 0

 0

 0

 0

 0

 .00345658321986

 .00345658321986

 .00345658321986

 .00345658321986

 .00345658321986

 0

 0

 0

 0

 0

 0

 0

 0

 0

 .140724835026646

 .131847700848376

 6.50727977374423E-02

 7.85587095423E-05

 7.85587095423E-05

 0

 0

 0

 .0537865297999

 .0537865297999

 0

 0

 0

 0

 0

 0

 0

 0

 .011207709228

 0

 .011207709228

 6.67749031109341E-02

 .00306378967215

 .00306378967215

 0

 0

 0

 0

 0

 0

 .0158950455641

 0

 0

 .0158950455641

 .0290405362941

 .0290405362941

 .00552529590447

 .00552529590447

 1.32502356761141E-02

 .0132240494396

 2.61862365141E-05

 .00887713417827

 .00887713417827

 0

 0

 0

 0

 0

 .00887713417827

 .00720121504137

 .0016759191369

 0

 0

 0

 0

 0

 0

 0

 0

 0

 0

 0

 0

 0

 0

 0

 0

 0

 0

 0

 0

 0

 0

 0

 0

 0

 0

 0

 0

 0

 0

 0

 0

 0

 4.5564051534523E-03

 0

 0

 0

 0

 0

 0

 0

 0

 0

 4.5564051534523E-03

 4.5564051534523E-03

 .00227820257673

 .00227820257673

 0

 0

 0

 0

 0

 .00219964386718

 .00219964386718

 0

 0

 7.85587095423E-05

 7.85587095423E-05

 0

 0

 0

 0

 0

 0

 0

 0

 5.6038546140146E-03

 0

 0

 0

 0

 0

 0

 0

 0

 0

 0

 0

 0

 0

 0

 0

 0

 0

 5.6038546140146E-03

 0

 0

 0

 0

 2.4876924688423E-03

 2.4876924688423E-03

 .0024091337593

 0

 2.61862365141E-05

 0

 0

 5.23724730282E-05

 0

 0

 0

 0

 0

 3.1161621451723E-03

 0

 0

 3.1161621451723E-03

 .00013093118257

 7.85587095423E-05

 .00290667225306

 .589007017911651

 .10267623337168

 .01008170105793

 .01008170105793

 0

 .00382319053106

 0

 0

 .00196396773856

 0

 0

 0

 0

 .00429454278831

 0

 0

 0

 0

 0

 2.61862365141E-05

 2.61862365141E-05

 0

 0

 0

 0

 0

 2.61862365141E-05

 0

 0

 0

 0

 0

 0

 0

 0

 0

 0

 0

 0

 0

 0

 0

 0

 0

 0

 0

 0

 0

 0

 0

 0

 0

 0

 0

 .00382319053106

 .00382319053106

 .00382319053106

 8.32722321147327E-02

 4.9230124646441E-03

 0

 2.61862365141E-05

 .00489682622813

 0

 0

 0

 0

 0

 0

 0

 0

 0

 0

 0

 0

 0

 0

 0

 3.5089556928841E-03

 0

 .00348276945637

 2.61862365141E-05

 0

 0

 2.61862365141E-05

 2.61862365141E-05

 0

 0

 0

 5.50172829160842E-02

 .000104744946056

 .054860165497

 0

 0

 0

 2.61862365141E-05

 2.61862365141E-05

 0

 0

 0

 0

 0

 0

 0

 0

 0

 1.97967948046062E-02

 .0114171991201

 2.61862365141E-05

 0

 2.61862365141E-05

 0

 0

 0

 0

 .000628469676338

 .00769875353514

 0

 0

 3.4827694563732E-03

 3.4827694563732E-03

 5.23724730282E-05

 .00314234838169

 .000288048601655

 0

 0

 0

 0

 0

 0

 0

 0

 0

 0

 0

 0

 0

 0

 0

 0

 0

 0

 0

 0

 0

 0

 0

 .00199015397507

 .00199015397507

 0

 0

 0

 0

 0

 0

 0

 0

 0

 .00199015397507

 0

 0

 0

 0

 0

 0

 6.7036765476071E-03

 .000916518277993

 .000916518277993

 .000916518277993

 .00356132816592

 0

 0

 0

 .00356132816592

 .00356132816592

 0

 0

 0

 0

 0

 0

 .00115219440662

 0

 0

 0

 0

 0

 0

 0

 0

 0

 0

 0

 0

 0

 0

 0

 .00115219440662

 .000183303655599

 .000968890751021

 0

 0

 0

 0

 0

 0

 0

 0

 0

 0

 0

 0

 .00104744946056

 .00104744946056

 .00104744946056

 2.61862365141E-05

 2.61862365141E-05

 2.61862365141E-05

 .479627107992364

 0

 0

 0

 0

 0

 0

 0

 0

 0

 0

 0

 0

 0

 0

 .326489996857952

 .323845186970032

 .000209489892113

 0

 0

 .0076201948256

 0

 .312637477742

 0

 0

 0

 0

 0

 0

 0

 0

 0

 0

 0

 0

 0

 .000288048601655

 .00306378967215

 0

 0

 0

 0

 0

 0

 0

 0

 2.61862365141E-05

 0

 0

 0

 0

 0

 0

 0

 0

 0

 0

 0

 0

 .00264480988792

 .00264480988792

 0

 0

 0

 0

 0

 0

 0

 0

 0

 0

 0

 0

 0

 0

 0

 0

 0

 .02126322404944

 .02126322404944

 .00013093118257

 .00748926364303

 0

 0

 0

 0

 .0061275793443

 0

 0

 .00751544987954

 0

 .00542055095842

 .00542055095842

 .00542055095842

 0

 0

 0

 0

 0

 .10322614433855

 .00343039698335

 .00343039698335

 .0997957473552

 .0997957473552

 0

 0

 0

 4.5564051534541E-03

 .00453021891694

 .00319472085472

 0

 .00133549806222

 0

 0

 2.61862365141E-05

 2.61862365141E-05

 0

 0

 0

 0

 0

 0

 0

 0

 0

 0

 0

 0

 0

 0

 .00879857546873

 .00879857546873

 .00879857546873

 0

 0

 0

 0

 0

 9.8722111658182E-03

 2.9852309626082E-03

 .00293285848958

 0

 0

 0

 5.23724730282E-05

 0

 .00688698020321

 0

 0

 0

 .00688698020321

 0

 0

 0

 0

 0

 0

 0

 0

 0

 0

 0

 0

 5.23724730282E-05

 5.23724730282E-05

 0

 0

 0

 0

 0

 0

 0

 0

 2.61862365141E-05

 2.61862365141E-05

 2.61862365141E-05

 2.61862365141E-05

 2.61862365141E-05

 2.61862365141E-05

 0

 0

 0

 0

 0

 0

 0

 0

 0

 0

 0

 0

 0

 0

 0

 0

 0

 0

 0

 0

 0

 0

 0

 0

 0

 0

 0

 0

 0

 0
