# Supplementary material for: The microbiome profiling of fungivorous black tinder fungus beetle Bolitophagus reticulatus reveals the insight into bacterial communities associated with larvae and adults
Source: PeerJ. 2019 May 7;7:e6852. doi: 10.7717/peerj.6852 (PMC6510215; doi:10.7717/peerj.6852)
Supplement: Data S1 — The first level represents the kingdom, the second level represents all phyla present in a particular sample; subsequent next levels represent the class, order, family and genus. [file peerj-07-6852-s003.zip › Supplemental_Data_S1/L-Betula-3.html]

Javascript must be enabled to view this page.

magnitude

 .999999999999948

 0

 0

 0

 0

 0

 0

 .999999999999948

 .000731775963974

 .000731775963974

 .000731775963974

 .000731775963974

 .000731775963974

 4.9254151421362E-03

 4.3202927103882E-03

 3.1381930762782E-03

 3.1381930762782E-03

 2.81452293836E-05

 0

 0

 0

 0

 1.40726146918E-05

 .000394033211371

 0

 1.40726146918E-05

 .00268786940614

 0

 .00118209963411

 .00118209963411

 .00118209963411

 0

 0

 0

 0

 0

 0

 0

 0

 0

 0

 0

 0

 0

 0

 0

 0

 0

 0

 0

 0

 0

 0

 0

 0

 0

 .000605122431748

 0

 0

 0

 .000605122431748

 .000605122431748

 .000605122431748

 .340360258935883

 5.7275541795672E-03

 5.7275541795672E-03

 .0056712637208

 .0056712637208

 5.62904587672E-05

 5.62904587672E-05

 0

 0

 0

 0

 0

 0

 0

 0

 0

 0

 0

 0

 0

 0

 .311244019138526

 0

 0

 0

 0

 0

 0

 0

 0

 0

 .075598086124349

 .03613847452853

 0

 .0146918097382

 .0148043906558

 .00664227413453

 0

 0

 .0255840135097

 .0255840135097

 .009738249366729

 0

 0

 0

 0

 .000379960596679

 .00935828877005

 0

 0

 .00413734871939

 .00413734871939

 1.54517309315958E-02

 0

 0

 .00116802701942

 .00116802701942

 0

 .00128060793695

 .00128060793695

 0

 .00586828032648

 .00586828032648

 .00379960596679

 .00379960596679

 .000281452293836

 .000281452293836

 0

 3.0537573881198E-03

 .00289895862651

 1.40726146918E-05

 .000140726146918

 1.40726146918E-05

 1.40726146918E-05

 0

 1.40726146918E-05

 .185364480720299

 2.81452293836E-05

 2.81452293836E-05

 0

 0

 0

 0

 0

 0

 0

 .00030959752322

 .00030959752322

 0

 0

 0

 0

 0

 0

 1.14269631297358E-02

 .0102870813397

 1.40726146918E-05

 .000267379679144

 .000211089220377

 .000647340275823

 .160976639459392

 .00316633830566

 0

 .000182943990994

 .00015479876161

 1.40726146918E-05

 8.44356881509E-05

 .000548831972981

 0

 .000112580917534

 0

 .0108781311568

 .140627638615

 .00123839009288

 2.81452293836E-05

 2.81452293836E-05

 0

 .000140726146918

 .00372924289333

 1.40726146918E-05

 0

 2.81452293836E-05

 0

 0

 1.26231353785672E-02

 0

 5.62904587672E-05

 0

 .0125668449198

 0

 0

 0

 0

 0

 0

 0

 0

 0

 .03171967351539

 .00349000844357

 0

 0

 0

 .00349000844357

 .02822966507182

 .0196735153392

 .00855614973262

 0

 .0030959752322

 .0030959752322

 0

 0

 0

 .0030959752322

 0

 0

 0

 0

 0

 0

 0

 .00681114551084

 .00681114551084

 .00681114551084

 .00681114551084

 0

 .00226569096538

 .00226569096538

 .00226569096538

 .00226569096538

 0

 0

 0

 0

 .00598086124402

 .00598086124402

 .00598086124402

 .00598086124402

 .00833098789755

 0

 0

 0

 0

 0

 0

 0

 .00833098789755

 0

 0

 0

 0

 0

 .00833098789755

 0

 0

 .00833098789755

 0

 0

 0

 0

 0

 0

 0

 0

 0

 0

 0

 0

 0

 0

 0

 0

 0

 0

 0

 0

 0

 0

 0

 0

 0

 0

 0

 0

 .03544891640868

 .03544891640868

 .00153391500141

 0

 0

 .00153391500141

 .00153391500141

 0

 0

 0

 0

 0

 0

 0

 0

 0

 0

 0

 0

 0

 0

 0

 0

 0

 0

 0

 0

 0

 0

 0

 0

 0

 .00495356037152

 0

 0

 0

 0

 0

 0

 .00495356037152

 .00495356037152

 0

 0

 0

 0

 .0170278637771

 0

 0

 .0170278637771

 0

 .0170278637771

 0

 0

 0

 0

 0

 .01193357725865

 0

 0

 0

 .01193357725865

 0

 .00593864339994

 .00599493385871

 0

 0

 0

 0

 0

 0

 0

 0

 0

 0

 0

 .000731775963974

 .000731775963974

 0

 0

 0

 0

 0

 0

 0

 .000731775963974

 .000450323670138

 .000450323670138

 .000281452293836

 .000281452293836

 0

 0

 0

 0

 0

 0

 0

 0

 0

 0

 0

 0

 0

 0

 0

 0

 0

 0

 0

 0

 0

 0

 0

 0

 0

 0

 0

 0

 0

 0

 0

 0

 0

 0

 0

 0

 0

 0

 .00754292147481

 .00754292147481

 .00754292147481

 .00754292147481

 .00754292147481

 0

 0

 0

 0

 0

 0

 0

 0

 0

 0

 0

 0

 0

 0

 0

 0

 0

 0

 0

 0

 0

 0

 0

 0

 0

 0

 9.62848297213945E-02

 9.62707571067027E-02

 8.26062482409509E-02

 8.44356881509E-05

 8.44356881509E-05

 .002237545736

 0

 .002237545736

 .0284970447509

 .0284970447509

 0

 0

 0

 0

 0

 0

 0

 0

 .0517872220659

 0

 .0517872220659

 1.36645088657518E-02

 0

 0

 0

 0

 1.40726146918E-05

 0

 0

 1.40726146918E-05

 0

 0

 0

 0

 .00544610188573

 .00544610188573

 .00472839853645

 .00472839853645

 .00347593582888

 0

 .00347593582888

 1.40726146918E-05

 1.40726146918E-05

 0

 0

 0

 0

 0

 1.40726146918E-05

 1.40726146918E-05

 0

 0

 0

 0

 0

 0

 0

 0

 0

 0

 0

 0

 0

 0

 0

 0

 0

 0

 0

 0

 0

 0

 0

 0

 0

 0

 0

 0

 .000745848578666

 .000745848578666

 .000745848578666

 .000745848578666

 .000745848578666

 0

 .010512243174782

 0

 0

 0

 0

 0

 0

 0

 0

 0

 .010512243174782

 .010512243174782

 .00676892766676

 .00676892766676

 0

 0

 0

 0

 0

 .00318041092035

 .00318041092035

 0

 0

 .000562904587672

 .000562904587672

 0

 0

 0

 0

 0

 0

 0

 0

 8.44356881509E-05

 0

 0

 0

 0

 0

 8.44356881509E-05

 8.44356881509E-05

 8.44356881509E-05

 8.44356881509E-05

 0

 0

 0

 0

 0

 0

 0

 0

 0

 0

 0

 0

 0

 0

 0

 0

 0

 0

 0

 0

 0

 0

 0

 0

 0

 0

 0

 0

 0

 0

 0

 0

 0

 .497143259217688

 .288179003658945

 .04066985645935

 .04066985645935

 0

 0

 0

 0

 0

 0

 0

 0

 .0350830284267

 .00558682803265

 0

 0

 0

 0

 0

 0

 0

 0

 0

 0

 0

 0

 0

 0

 0

 0

 0

 0

 0

 0

 0

 0

 0

 0

 0

 0

 0

 1.40726146918E-05

 1.40726146918E-05

 1.40726146918E-05

 0

 0

 0

 0

 0

 0

 0

 0

 0

 0

 0

 0

 0

 .139769209119068

 .00505206867436

 0

 .00505206867436

 0

 0

 0

 0

 0

 0

 0

 0

 0

 .00015479876161

 .00015479876161

 1.40726146918E-05

 1.40726146918E-05

 0

 0

 0

 0

 0

 0

 0

 0

 0

 .00243456234168

 .00243456234168

 0

 0

 0

 .082198142414899

 .000225161835069

 .0737686462145

 0

 0

 0

 .00820433436533

 0

 0

 0

 0

 0

 0

 0

 0

 1.40726146918E-05

 1.40726146918E-05

 4.99014916971358E-02

 .0168449197861

 .00536166619758

 0

 .000576977202364

 0

 1.40726146918E-05

 0

 0

 0

 .0271038558964

 0

 0

 7.46552209400704E-02

 7.46552209400704E-02

 4.22178440754E-05

 .00256121587391

 .000197016605685

 0

 0

 .0718547706164

 0

 0

 0

 0

 0

 0

 0

 0

 0

 0

 0

 0

 0

 0

 0

 0

 0

 0

 0

 0

 3.30706445257644E-02

 3.30706445257644E-02

 .000182943990994

 0

 0

 0

 2.81452293836E-05

 0

 0

 0

 1.40726146918E-05

 .0324936673234

 0

 0

 .000351815367295

 0

 0

 0

 0

 0

 0

 0

 0

 0

 0

 0

 0

 0

 0

 0

 0

 0

 0

 0

 0

 0

 0

 0

 0

 0

 0

 0

 0

 0

 0

 0

 0

 0

 0

 0

 0

 0

 0

 0

 0

 0

 0

 0

 0

 0

 0

 0

 0

 0

 0

 0

 0

 0

 0

 0

 0

 .208964255558743

 0

 0

 0

 0

 0

 0

 0

 0

 0

 0

 0

 0

 0

 0

 8.89811426963196E-02

 8.73346467773796E-02

 .00564311849142

 0

 0

 .000126653532226

 0

 .0692513368984

 0

 0

 0

 0

 0

 0

 0

 0

 2.81452293836E-05

 .00109766394596

 0

 0

 0

 0

 0

 0

 .0089361103293

 0

 0

 0

 0

 0

 0

 0

 0

 0

 .00225161835069

 0

 0

 0

 0

 0

 0

 0

 0

 0

 0

 0

 .00164649591894

 .00164649591894

 0

 0

 0

 0

 0

 0

 0

 0

 0

 0

 0

 0

 0

 0

 0

 1.90402476780072E-02

 1.90402476780072E-02

 .00439065578384

 0

 0

 0

 1.40726146918E-05

 .00174500422178

 .00329299183788

 4.22178440754E-05

 0

 .00955530537574

 0

 0

 0

 0

 0

 0

 0

 0

 0

 .0588938924852

 0

 0

 .0588938924852

 .0588938924852

 0

 0

 0

 1.78300028145772E-02

 4.3484379397772E-03

 .00136504362511

 0

 5.62904587672E-05

 .0029271038559

 0

 .0134815648748

 .0134815648748

 0

 0

 0

 0

 0

 0

 0

 0

 0

 0

 0

 0

 0

 0

 .00239234449761

 .00239234449761

 .00239234449761

 0

 0

 0

 0

 0

 .021826625387029

 .018167745567159

 5.62904587672E-05

 0

 1.40726146918E-05

 .0180973824937

 0

 0

 .00365887981987

 0

 0

 0

 0

 .00365887981987

 0

 0

 0

 0

 0

 0

 .00213903743316

 .00213903743316

 .00213903743316

 .00213903743316

 .00213903743316

 .00334928229665

 .00334928229665

 .00201238390093

 0

 0

 0

 0

 0

 .00201238390093

 .00201238390093

 0

 0

 0

 0

 0

 0

 0

 0

 0

 0

 0

 0

 0

 0

 0

 0

 0

 .00133689839572

 .00133689839572

 .00133689839572

 0

 0

 0

 0

 0

 0

 0

 0

 0

 0

 0

 0

 0

 0

 0

 0
