# Supplementary material for: The microbiome profiling of fungivorous black tinder fungus beetle Bolitophagus reticulatus reveals the insight into bacterial communities associated with larvae and adults
Source: PeerJ. 2019 May 7;7:e6852. doi: 10.7717/peerj.6852 (PMC6510215; doi:10.7717/peerj.6852)
Supplement: Data S1 — The first level represents the kingdom, the second level represents all phyla present in a particular sample; subsequent next levels represent the class, order, family and genus. [file peerj-07-6852-s003.zip › Supplemental_Data_S1/L-Fagus-1.html]

Javascript must be enabled to view this page.

magnitude

 .999999999999979

 0

 0

 0

 0

 0

 0

 .999999999999979

 0

 0

 0

 0

 0

 2.48822372795064E-02

 1.56309677054264E-02

 1.56309677054264E-02

 1.56309677054264E-02

 5.65826885264E-05

 0

 .00780841101665

 0

 0

 .00776597400025

 0

 0

 0

 0

 0

 0

 0

 0

 0

 .00507829629525

 0

 0

 0

 0

 0

 .00507829629525

 .00507829629525

 .00507829629525

 0

 0

 0

 0

 0

 0

 0

 0

 0

 0

 0

 0

 0

 0

 0

 .00417297327883

 .00417297327883

 .00417297327883

 .00417297327883

 0

 0

 0

 .385073486766663

 9.1946868855481E-03

 9.1946868855481E-03

 .00912395852489

 .00912395852489

 7.07283606581E-05

 7.07283606581E-05

 0

 0

 0

 0

 0

 0

 0

 0

 0

 0

 0

 0

 0

 0

 .35894643033958

 0

 0

 0

 0

 0

 0

 0

 0

 0

 6.21136463298361E-02

 .02946543505012

 0

 .00647871783628

 .00292815413124

 .0200585630826

 0

 0

 .0253490444598

 .0253490444598

 7.2001471149948E-03

 0

 .00123067347545

 0

 0

 4.24370163948E-05

 .00592703662315

 0

 0

 9.90197049213E-05

 9.90197049213E-05

 .020454641902308

 0

 0

 0

 0

 0

 .00301302816403

 .00301302816403

 0

 .00488025688541

 .00488025688541

 .0081903441642

 .0081903441642

 0

 0

 0

 .004371012688668

 .00430028432801

 5.65826885264E-05

 1.41456721316E-05

 1.41456721316E-05

 1.41456721316E-05

 0

 1.41456721316E-05

 4.5690520985148E-03

 0

 0

 0

 0

 0

 0

 .0021925791804

 0

 .0021925791804

 0

 0

 0

 0

 0

 0

 0

 0

 0

 0

 0

 0

 0

 0

 1.41456721316E-05

 0

 0

 0

 0

 0

 0

 0

 0

 0

 0

 0

 0

 0

 0

 0

 0

 0

 1.41456721316E-05

 0

 0

 0

 0

 0

 2.3623272459832E-03

 1.41456721316E-05

 0

 1.41456721316E-05

 .00233403590172

 0

 0

 0

 0

 0

 0

 0

 0

 0

 .267027852828439

 .24958623909018

 .0059977649838

 0

 .00401737088538

 .239571103221

 .017441613738259

 .0171021176071

 .000339496131159

 0

 0

 0

 0

 0

 0

 0

 .00476709150835

 .00476709150835

 0

 0

 0

 0

 .00476709150835

 0

 0

 0

 0

 0

 0

 0

 0

 0

 0

 0

 0

 0

 .00906737583636

 .00906737583636

 .00906737583636

 .00906737583636

 .007864993705175

 .004286138655875

 0

 0

 .004286138655875

 .00345154400011

 0

 .000834594655765

 .0035788550493

 0

 0

 0

 0

 0

 .0035788550493

 0

 0

 0

 0

 .0035788550493

 .00182479170498

 .00182479170498

 .00182479170498

 .00182479170498

 .00182479170498

 0

 0

 0

 0

 0

 0

 0

 0

 0

 0

 0

 1.41456721316E-05

 1.41456721316E-05

 1.41456721316E-05

 1.41456721316E-05

 1.41456721316E-05

 0

 0

 0

 0

 0

 2.29867172138687E-02

 2.29867172138687E-02

 7.07283606581E-05

 0

 0

 7.07283606581E-05

 0

 7.07283606581E-05

 0

 0

 0

 0

 0

 4.4700323935916E-03

 0

 0

 0

 0

 1.41456721316E-05

 0

 0

 0

 0

 0

 0

 0

 0

 0

 1.41456721316E-05

 0

 .00445588672146

 .00445588672146

 0

 0

 0

 0

 0

 0

 0

 0

 0

 0

 0

 0

 0

 .01496612111524

 0

 0

 .00940687196752

 0

 .00940687196752

 .00555924914772

 .00555924914772

 0

 0

 0

 .003479835344379

 0

 0

 0

 0

 0

 0

 0

 0

 0

 .003479835344379

 .00314033921322

 0

 0

 .000339496131159

 0

 0

 0

 0

 0

 0

 0

 0

 0

 0

 0

 0

 0

 0

 0

 0

 0

 0

 0

 0

 0

 0

 0

 0

 0

 0

 0

 0

 0

 0

 0

 0

 0

 0

 0

 0

 0

 0

 0

 0

 0

 0

 0

 0

 0

 0

 0

 0

 0

 0

 0

 0

 0

 0

 0

 0

 0

 0

 0

 0

 0

 0

 0

 0

 0

 0

 0

 0

 0

 0

 0

 0

 0

 .00430028432801

 .00430028432801

 .00430028432801

 .00430028432801

 .00430028432801

 0

 0

 0

 0

 0

 0

 0

 0

 0

 6.95259785268781E-02

 6.43345168545781E-02

 4.47993436408481E-02

 0

 0

 1.69465152136948E-02

 4.24370163948E-05

 .0169040781973

 .00775182832812

 .00775182832812

 0

 0

 0

 7.0445447215333E-03

 .00442759537719

 .00223501619679

 9.90197049213E-05

 .000282913442632

 .0130564553775

 0

 .0130564553775

 .01953517321373

 0

 0

 0

 0

 .00306961085256

 0

 .00306961085256

 0

 0

 0

 0

 0

 .0140607980988

 .0140607980988

 0

 0

 .00240476426237

 0

 .00240476426237

 .0051914616723

 .0051914616723

 0

 0

 0

 0

 0

 0

 0

 0

 0

 0

 .0051914616723

 0

 .0051914616723

 0

 0

 0

 0

 0

 0

 0

 0

 0

 0

 0

 0

 0

 0

 0

 0

 0

 0

 0

 0

 0

 0

 0

 0

 0

 0

 0

 0

 8.9966474757032E-03

 0

 0

 0

 0

 0

 0

 0

 0

 0

 8.6430056724132E-03

 8.6430056724132E-03

 .00861471432815

 .00861471432815

 0

 0

 0

 0

 0

 1.41456721316E-05

 1.41456721316E-05

 0

 0

 1.41456721316E-05

 1.41456721316E-05

 0

 0

 0

 0

 .00035364180329

 .00035364180329

 .00035364180329

 .00035364180329

 7.9074307215736E-03

 0

 0

 0

 0

 0

 0

 0

 0

 0

 0

 0

 0

 0

 0

 0

 0

 0

 7.9074307215736E-03

 .00295644547551

 .00295644547551

 0

 .00295644547551

 4.7388001640896E-03

 4.7388001640896E-03

 .0046539261313

 0

 2.82913442632E-05

 0

 0

 5.65826885264E-05

 .000212185081974

 .000212185081974

 0

 0

 .000212185081974

 0

 0

 0

 0

 0

 0

 0

 .473257606835215

 .28397436804206

 .023156465279501

 .023156465279501

 .00386176849193

 .00751135190189

 0

 0

 .000707283606581

 0

 0

 0

 0

 .0110760612791

 0

 0

 0

 0

 0

 5.7997255739616E-03

 5.7997255739616E-03

 1.41456721316E-05

 0

 .00212185081974

 0

 .00220672485253

 .00145700422956

 0

 0

 0

 0

 0

 0

 .00128725616398

 .00128725616398

 .00128725616398

 0

 0

 0

 0

 0

 .00342325265585

 .00342325265585

 .00342325265585

 0

 0

 0

 0

 0

 0

 0

 0

 0

 0

 .00906737583636

 .00906737583636

 .00906737583636

 .220021784335014

 9.4776003281832E-03

 0

 0

 1.41456721316E-05

 0

 1.41456721316E-05

 0

 0

 0

 0

 .0035788550493

 .00587045393462

 0

 0

 2.57026862630898E-02

 .0171587002956

 4.24370163948E-05

 .00810547013141

 .000396078819685

 .00200868544269

 0

 .00200868544269

 0

 0

 0

 .0319126363289

 .0319126363289

 0

 0

 0

 5.86055196412877E-02

 .00275840606566

 .0361421922963

 7.07283606581E-05

 0

 0

 .00916639554128

 0

 2.82913442632E-05

 .00782255668878

 1.41456721316E-05

 4.24370163948E-05

 .00256036665582

 0

 0

 1.41456721316E-05

 1.41456721316E-05

 9.23005106587313E-02

 .0415458390505

 .0394664252472

 0

 .00140042154103

 0

 9.90197049213E-05

 0

 0

 0

 .00278669740993

 0

 .00700210770515

 1.41456721316E-05

 1.41456721316E-05

 0

 0

 0

 0

 0

 1.41456721316E-05

 0

 0

 0

 0

 0

 0

 0

 0

 0

 .000806303311502

 0

 0

 .000806303311502

 .000806303311502

 0

 0

 0

 0

 0

 0

 .02039805921376

 .02039805921376

 0

 .00338081563946

 0

 0

 0

 0

 0

 0

 0

 .0170172435743

 0

 0

 0

 0

 0

 0

 .003295941606664

 0

 0

 0

 0

 0

 0

 0

 0

 0

 .000226330754106

 .000226330754106

 .000226330754106

 0

 0

 0

 .003069610852558

 0

 0

 0

 0

 0

 0

 0

 0

 0

 .00212185081974

 0

 .00212185081974

 0

 0

 0

 .000947760032818

 0

 .000947760032818

 0

 0

 0

 0

 0

 0

 0

 0

 0

 0

 0

 0

 0

 0

 0

 0

 0

 0

 .185987297186491

 .00381933147554

 .00381933147554

 .00381933147554

 0

 0

 0

 0

 0

 0

 0

 0

 0

 0

 0

 9.18337034784176E-02

 8.20166070190756E-02

 .0123067347545

 0

 0

 .00207941380335

 0

 .0518721797066

 0

 1.41456721316E-05

 0

 0

 0

 0

 .00260280367222

 0

 0

 .00444174104933

 0

 0

 .00298473681977

 .000381933147554

 .00533291839362

 0

 0

 0

 0

 0

 0

 0

 0

 0

 0

 0

 0

 0

 0

 0

 .00541779242641

 0

 .00541779242641

 0

 0

 0

 0

 0

 0

 0

 0

 0

 .004399304032932

 .000367787475422

 .00403151655751

 0

 0

 0

 0

 0

 0

 .00316863055748

 .00316863055748

 .00316863055748

 0

 9.2795609183416E-03

 9.2795609183416E-03

 .00297059114764

 0

 0

 0

 0

 1.41456721316E-05

 0

 0

 0

 .00629482409857

 0

 .00257451232795

 .00257451232795

 .00257451232795

 0

 0

 .00983124213147

 .00983124213147

 .00983124213147

 .0299605335748

 0

 0

 .0299605335748

 .0299605335748

 0

 0

 0

 .0118540732463

 0

 0

 0

 0

 0

 0

 .0118540732463

 .0118540732463

 .00169748065579

 .00169748065579

 .00169748065579

 0

 0

 0

 0

 0

 0

 0

 0

 0

 0

 0

 .00490854822967

 .00490854822967

 .00490854822967

 0

 0

 0

 0

 0

 1.70596805907316E-02

 1.50792864923016E-02

 0

 0

 0

 .00588459960675

 .00918054121342

 1.41456721316E-05

 .00198039409843

 0

 0

 0

 .00198039409843

 0

 0

 0

 0

 0

 0

 0

 0

 0

 0

 0

 0

 .00123067347545

 .00123067347545

 0

 0

 0

 0

 0

 0

 0

 0

 0

 0

 0

 .00123067347545

 .00123067347545

 0

 0

 .00123067347545

 0

 0

 0

 0

 0

 0

 0

 0

 0

 0

 0

 0

 0

 0

 0

 0

 0

 0

 0

 0

 0

 0

 0

 0

 0

 0

 0

 0
