# Supplementary material for: The microbiome profiling of fungivorous black tinder fungus beetle Bolitophagus reticulatus reveals the insight into bacterial communities associated with larvae and adults
Source: PeerJ. 2019 May 7;7:e6852. doi: 10.7717/peerj.6852 (PMC6510215; doi:10.7717/peerj.6852)
Supplement: Data S1 — The first level represents the kingdom, the second level represents all phyla present in a particular sample; subsequent next levels represent the class, order, family and genus. [file peerj-07-6852-s003.zip › Supplemental_Data_S1/L-Fagus-2.html]

Javascript must be enabled to view this page.

magnitude

 .99999999999988

 0

 0

 0

 0

 0

 0

 .99999999999988

 .00146208124086

 .00146208124086

 .00146208124086

 .00146208124086

 .00146208124086

 .00368698747695

 0

 0

 0

 0

 0

 0

 0

 0

 0

 0

 0

 0

 0

 0

 0

 0

 0

 0

 .00264869790011

 .00264869790011

 .00264869790011

 .00264869790011

 0

 0

 0

 0

 0

 0

 0

 0

 0

 0

 0

 0

 0

 0

 0

 0

 .00103828957684

 .00103828957684

 .00103828957684

 .00103828957684

 0

 0

 0

 0

 0

 0

 0

 .410145572436514

 .00620854787786

 .00046617083042

 .00046617083042

 .00046617083042

 0

 0

 .00574237704744

 0

 0

 .00574237704744

 0

 0

 .00574237704744

 0

 0

 0

 0

 0

 0

 0

 .387027737164344

 0

 0

 0

 0

 0

 0

 0

 0

 0

 .132180620007115

 .0519568580086

 0

 0

 .0370393914352

 .0149174665734

 0

 0

 .0389040747568

 .0389040747568

 .04119254974251

 0

 .00656877079228

 0

 0

 .00544572288263

 .0291780560676

 0

 0

 .000127137499205

 .000127137499205

 3.26955268790018E-02

 0

 0

 0

 0

 0

 .0206810332041

 .0206810332041

 0

 0

 0

 .0119721145085

 .0119721145085

 0

 0

 0

 4.23791664018E-05

 0

 0

 4.23791664018E-05

 0

 0

 0

 0

 .136121882482503

 0

 0

 0

 0

 0

 0

 0

 0

 0

 0

 0

 2.11895832009E-05

 0

 2.11895832009E-05

 0

 0

 0

 2.9453520649218E-03

 .00290297289852

 2.11895832009E-05

 2.11895832009E-05

 0

 0

 .104718920178786

 .0593944017121

 0

 4.23791664018E-05

 .000127137499205

 0

 .00046617083042

 2.11895832009E-05

 0

 4.23791664018E-05

 0

 0

 .0307248956413

 .0125654228381

 .000614497912826

 2.11895832009E-05

 2.11895832009E-05

 4.23791664018E-05

 .00046617083042

 2.11895832009E-05

 4.23791664018E-05

 4.23791664018E-05

 6.35687496027E-05

 0

 .028436420655594

 .00286059373212

 .000105947916004

 .00925984785879

 .00860297077956

 0

 0

 .00760706036912

 0

 0

 0

 0

 0

 0

 .06159811836507

 .03667916852077

 .0071196999555

 .0233297311042

 .00300892081453

 .00322081664654

 .0249189498443

 .0249189498443

 0

 0

 .00830631661475

 .00830631661475

 0

 0

 0

 .00830631661475

 .016125272815904

 .016125272815904

 0

 0

 .000105947916004

 0

 .0160193248999

 .00349628122815

 .00349628122815

 .00349628122815

 0

 .00349628122815

 0

 0

 0

 0

 0

 0

 0

 0

 .00760706036912

 .00760706036912

 .00760706036912

 .00760706036912

 .00580594579704

 0

 0

 0

 0

 0

 0

 0

 .00580594579704

 0

 0

 0

 0

 0

 .00580594579704

 0

 0

 .00286059373212

 0

 .00294535206492

 0

 0

 0

 0

 0

 0

 0

 0

 0

 0

 0

 0

 0

 0

 0

 0

 0

 0

 0

 0

 0

 0

 0

 0

 0

 0

 .04655351429237

 .04655351429237

 .00360222914415

 0

 0

 0

 0

 0

 .00360222914415

 .00360222914415

 0

 0

 0

 .00334795414574

 0

 0

 0

 0

 .00334795414574

 0

 0

 0

 0

 0

 0

 0

 0

 0

 .00334795414574

 0

 0

 0

 0

 0

 0

 0

 0

 0

 0

 0

 0

 0

 0

 0

 0

 .01565910198548

 0

 0

 .01565910198548

 .00188587290488

 .0137732290806

 0

 0

 0

 0

 0

 .023944229017

 0

 0

 0

 .023944229017

 0

 0

 .023944229017

 0

 0

 0

 0

 0

 0

 0

 0

 0

 0

 0

 .00419553747378

 .00419553747378

 0

 0

 0

 0

 0

 0

 0

 .00419553747378

 0

 0

 0

 0

 .00419553747378

 .00419553747378

 0

 0

 0

 0

 0

 0

 0

 0

 0

 0

 0

 0

 .00347509164495

 0

 0

 0

 0

 0

 0

 0

 0

 0

 .00347509164495

 .00347509164495

 .00347509164495

 .00347509164495

 0

 0

 0

 0

 0

 0

 0

 0

 0

 0

 .00752230203632

 .00752230203632

 .00752230203632

 .00752230203632

 .00752230203632

 0

 0

 0

 0

 0

 0

 0

 0

 0

 0

 0

 0

 .00355984997775

 .00355984997775

 .00355984997775

 .00355984997775

 .00355984997775

 0

 0

 0

 0

 0

 0

 0

 0

 0

 7.46932807832268E-02

 6.46706079291968E-02

 5.52200538216018E-02

 4.23791664018E-05

 4.23791664018E-05

 0

 0

 0

 .0320810289662

 .0320810289662

 0

 0

 0

 0

 0

 0

 0

 0

 .023096645689

 0

 .023096645689

 .009450554107595

 0

 0

 0

 0

 0

 0

 0

 0

 .006780666624285

 .000614497912826

 .00572118746424

 .000444981247219

 0

 0

 0

 0

 .00266988748331

 0

 .00266988748331

 .00675947704109

 .00675947704109

 .00394126247537

 0

 .00394126247537

 0

 0

 0

 0

 0

 0

 0

 0

 0

 0

 .00281821456572

 .00281821456572

 0

 0

 0

 0

 0

 0

 0

 .00326319581294

 .00326319581294

 0

 0

 .00326319581294

 0

 .00326319581294

 0

 0

 0

 0

 0

 0

 0

 0

 0

 0

 0

 0

 2.24397686097369E-02

 0

 0

 0

 0

 0

 0

 0

 0

 0

 2.24397686097369E-02

 2.24397686097369E-02

 .0022672854025

 .0022672854025

 .0169516665607

 0

 .0169516665607

 .00281821456572

 .00281821456572

 2.11895832009E-05

 2.11895832009E-05

 0

 0

 .000381412497616

 .000381412497616

 0

 0

 0

 0

 0

 0

 0

 0

 .00379293539296

 0

 0

 0

 0

 0

 0

 0

 0

 0

 0

 0

 0

 0

 0

 0

 0

 0

 .00379293539296

 0

 0

 0

 0

 0

 0

 0

 0

 0

 0

 0

 0

 .00379293539296

 .00379293539296

 0

 .00379293539296

 0

 0

 0

 0

 0

 0

 0

 0

 .409382747441272

 .255567562985965

 .00853940202996

 .00853940202996

 0

 0

 0

 0

 0

 0

 0

 0

 0

 .00853940202996

 0

 0

 0

 0

 0

 5.2762062170309E-03

 5.2762062170309E-03

 0

 0

 .00275464581612

 0

 .00250037081771

 2.11895832009E-05

 0

 0

 0

 0

 0

 0

 0

 0

 0

 0

 0

 0

 0

 0

 2.11895832009E-05

 2.11895832009E-05

 2.11895832009E-05

 0

 0

 0

 0

 0

 0

 0

 0

 0

 0

 0

 0

 0

 .227321848579197

 .00974720827241

 0

 0

 .00974720827241

 0

 0

 0

 0

 0

 0

 0

 0

 0

 0

 3.93702455872409E-02

 .0290509185684

 .00252156040091

 .00777657703473

 2.11895832009E-05

 .00794609370034

 .00794609370034

 0

 0

 0

 0

 0

 0

 0

 0

 0

 9.62007077320675E-02

 .000296654164813

 .079312609921

 0

 8.47583328036E-05

 0

 .0129680249189

 .00351747081135

 0

 0

 0

 0

 2.11895832009E-05

 0

 0

 0

 0

 .074057593287139

 .0145996228254

 .0540122475791

 .000402602080817

 .00156802915687

 0

 .000169516665607

 0

 0

 0

 .000826393744835

 .00247918123451

 0

 .001038289576844

 .001038289576844

 0

 0

 .000402602080817

 .000635687496027

 0

 0

 0

 0

 0

 0

 0

 0

 0

 0

 0

 .000762824995232

 0

 0

 .000762824995232

 .000762824995232

 0

 0

 0

 0

 0

 0

 .0126078020045

 .0126078020045

 0

 0

 0

 0

 0

 0

 0

 0

 0

 .0126078020045

 0

 0

 0

 0

 0

 0

 6.3144957938636E-03

 0

 0

 0

 8.47583328036E-05

 8.47583328036E-05

 0

 8.47583328036E-05

 0

 0

 0

 0

 0

 0

 0

 0

 0

 0

 0

 0

 0

 0

 0

 0

 0

 0

 0

 0

 0

 0

 0

 0

 0

 0

 0

 0

 0

 .00622973746106

 .00622973746106

 .00622973746106

 0

 0

 0

 0

 0

 0

 0

 0

 0

 0

 0

 0

 0

 .147500688661444

 0

 0

 0

 0

 0

 0

 0

 0

 0

 0

 0

 0

 0

 0

 3.02587248108967E-02

 3.02587248108967E-02

 .00898438327718

 0

 0

 .000211895832009

 0

 .00822155828195

 2.11895832009E-05

 0

 4.23791664018E-05

 0

 0

 0

 0

 0

 0

 0

 0

 0

 0

 0

 0

 0

 .0114847540949

 0

 0

 .000190706248808

 .000254274998411

 0

 .000360222914415

 0

 0

 0

 .000487360413621

 0

 0

 0

 0

 0

 0

 0

 0

 0

 0

 0

 0

 0

 0

 0

 0

 0

 0

 0

 0

 0

 0

 0

 0

 0

 0

 0

 0

 9.9379145212209E-03

 9.9379145212209E-03

 0

 0

 0

 0

 .00281821456572

 2.11895832009E-05

 0

 0

 0

 .0070985103723

 0

 0

 0

 0

 0

 0

 .0069925624563

 .0069925624563

 .0069925624563

 .0546055559087

 0

 0

 .0546055559087

 .0546055559087

 0

 0

 0

 .010128620770034

 .005954272879454

 .00440743330579

 .00144089165766

 .000105947916004

 0

 0

 .00417434789058

 .00417434789058

 0

 0

 0

 0

 0

 0

 0

 0

 0

 0

 0

 0

 0

 0

 .0105100332676

 .0105100332676

 .0105100332676

 0

 0

 0

 0

 0

 .025067276926692

 .00578475621385

 0

 0

 0

 .00256393956731

 0

 .00322081664654

 .019282520712842

 .000529739580022

 0

 0

 0

 .0159981353167

 .00275464581612

 0

 0

 0

 0

 0

 0

 0

 0

 0

 0

 .00909033119319

 .00909033119319

 0

 0

 0

 0

 0

 0

 0

 0

 0

 0

 0

 0

 0

 0

 0

 0

 0

 0

 0

 0

 0

 0

 0

 0

 0

 .00909033119319

 .00909033119319

 .00909033119319

 0

 0

 0

 0

 0

 0

 0

 0

 0

 0

 0

 0

 0

 0

 0

 0
