# Supplementary material for: The microbiome profiling of fungivorous black tinder fungus beetle Bolitophagus reticulatus reveals the insight into bacterial communities associated with larvae and adults
Source: PeerJ. 2019 May 7;7:e6852. doi: 10.7717/peerj.6852 (PMC6510215; doi:10.7717/peerj.6852)
Supplement: Data S1 — The first level represents the kingdom, the second level represents all phyla present in a particular sample; subsequent next levels represent the class, order, family and genus. [file peerj-07-6852-s003.zip › Supplemental_Data_S1/L-Fagus-3.html]

Javascript must be enabled to view this page.

magnitude

 1.00000000000035

 0

 0

 0

 0

 0

 0

 1.00000000000035

 .000553755281185

 .000553755281185

 .000553755281185

 .000553755281185

 .000553755281185

 3.6301735099917E-03

 1.7433036629917E-03

 1.7433036629917E-03

 1.7433036629917E-03

 0

 0

 2.05094548587E-05

 0

 0

 0

 .00145617129497

 0

 0

 .000266622913163

 0

 0

 0

 0

 0

 .001886869847

 0

 0

 0

 0

 0

 .001886869847

 .001886869847

 0

 .001886869847

 0

 0

 0

 0

 0

 0

 0

 0

 0

 0

 0

 0

 0

 0

 0

 0

 0

 0

 0

 0

 0

 .306083104310987

 2.5636818573407E-03

 2.05094548587E-05

 2.05094548587E-05

 2.05094548587E-05

 0

 0

 .002543172402482

 0

 0

 .002543172402482

 0

 .000758849829772

 .00178432257271

 0

 0

 0

 0

 0

 0

 0

 .297079453628016

 .00205094548587

 .00205094548587

 .00205094548587

 0

 0

 0

 0

 0

 0

 2.22322490668137E-02

 .0081422535789

 .000533245826326

 .00475819352722

 .00244062512818

 .000410189097174

 0

 0

 .00479921243693

 .00479921243693

 .009270273596125

 .000697321465195

 0

 0

 0

 .00514787316953

 .0034250789614

 0

 0

 2.05094548587E-05

 2.05094548587E-05

 5.5785717215727E-03

 0

 0

 0

 0

 0

 .00147668074983

 .00147668074983

 0

 0

 0

 .00319947495796

 .00319947495796

 0

 0

 0

 9.024160137827E-04

 .000881906558924

 0

 2.05094548587E-05

 0

 0

 0

 0

 .210139874482037

 0

 0

 0

 0

 0

 0

 1.2100578366661E-03

 6.15283645761E-05

 .00114852947209

 0

 0

 .000922925468641

 0

 .000922925468641

 0

 0

 0

 5.537552811851E-04

 .000492226916609

 0

 0

 6.15283645761E-05

 0

 .184277451905225

 .0848271052955

 2.05094548587E-05

 6.15283645761E-05

 .000430698552032

 0

 .000615283645761

 .00123056729152

 .00196890766643

 .000123056729152

 6.15283645761E-05

 .00047171746175

 .0799253455843

 .0103572747036

 .000799868739489

 .000635793100619

 4.10189097174E-05

 8.20378194348E-05

 .00229705894417

 .000102547274293

 6.15283645761E-05

 8.20378194348E-05

 8.20378194348E-05

 0

 .02317568399032

 .00771155502687

 .00131260511096

 .00572213790557

 .00842938594692

 0

 0

 0

 0

 0

 0

 0

 0

 0

 .042044382460344

 .031543541572697

 .000205094548587

 .0164485827967

 .0069527051971

 .00793715903031

 .010500840887647

 .00982402887731

 .000676812010337

 0

 .00381475860372

 .00381475860372

 0

 0

 0

 .00381475860372

 1.12186718076587E-02

 1.12186718076587E-02

 0

 0

 2.05094548587E-05

 0

 .0111981623528

 0

 0

 0

 0

 0

 0

 0

 0

 0

 .00176381311785

 .00176381311785

 .00176381311785

 .00176381311785

 .000553755281185

 .000553755281185

 .000553755281185

 .000553755281185

 4.1224004265947E-03

 7.998687394887E-04

 .00077935928463

 .00077935928463

 2.05094548587E-05

 2.05094548587E-05

 0

 0

 .003322531687106

 .000410189097174

 .000410189097174

 0

 0

 0

 .002912342589932

 .000984453833217

 0

 .00137413347553

 0

 .000553755281185

 0

 0

 0

 0

 0

 0

 0

 0

 0

 0

 0

 0

 0

 0

 0

 0

 0

 0

 0

 0

 0

 0

 0

 0

 0

 0

 5.06378440460437E-02

 5.06378440460437E-02

 0

 0

 0

 0

 0

 0

 0

 0

 0

 0

 0

 .004573608433485

 0

 0

 0

 0

 .004573608433485

 .00262521022191

 0

 0

 0

 .000922925468641

 .000389679642315

 0

 0

 0

 .000635793100619

 0

 0

 0

 0

 0

 0

 0

 0

 0

 0

 0

 0

 0

 0

 0

 0

 .010008613971

 0

 0

 .010008613971

 0

 .010008613971

 0

 0

 0

 0

 0

 3.60556216415587E-02

 0

 0

 0

 .0360351121867

 0

 .0068501579228

 .0291849542639

 0

 0

 2.05094548587E-05

 2.05094548587E-05

 0

 0

 0

 0

 0

 0

 0

 2.256040034457E-04

 2.256040034457E-04

 .000205094548587

 .000205094548587

 0

 .000205094548587

 2.05094548587E-05

 2.05094548587E-05

 2.05094548587E-05

 0

 0

 0

 0

 0

 0

 0

 0

 0

 0

 0

 0

 0

 0

 0

 0

 0

 0

 0

 .000328151277739

 .000328151277739

 0

 0

 0

 0

 0

 .000328151277739

 .000328151277739

 .000328151277739

 0

 0

 0

 0

 0

 0

 0

 0

 0

 0

 0

 0

 0

 0

 0

 0

 0

 0

 0

 .00110751056237

 .00110751056237

 .00110751056237

 0

 0

 .00110751056237

 .00110751056237

 0

 0

 0

 0

 0

 0

 0

 0

 0

 0

 0

 0

 0

 0

 0

 0

 0

 0

 0

 8.94417326387506E-02

 8.48065958406906E-02

 6.53636326346516E-02

 8.20378194348E-05

 8.20378194348E-05

 8.20378194348E-05

 0

 8.20378194348E-05

 .0430903646581

 .0430903646581

 0

 0

 0

 .000902416013782

 0

 .000902416013782

 0

 0

 .0212067763239

 0

 .0212067763239

 .019442963206039

 0

 0

 .000799868739489

 .000799868739489

 .00200992657615

 0

 0

 .00200992657615

 0

 0

 0

 0

 .00324049386767

 .00324049386767

 .00461462734321

 .00461462734321

 .00877804667952

 .00383526805858

 .00494277862094

 .003896796423147

 .003896796423147

 0

 0

 0

 .000676812010337

 .000676812010337

 .00321998441281

 0

 .00321998441281

 0

 0

 0

 0

 0

 0

 0

 0

 0

 0

 .000738340374913

 .000738340374913

 .000738340374913

 .000738340374913

 0

 0

 0

 0

 0

 0

 0

 0

 0

 0

 0

 0

 0

 0

 0

 0

 0

 0

 0

 1.46027318593857E-02

 0

 0

 0

 0

 0

 0

 0

 0

 0

 1.46027318593857E-02

 1.46027318593857E-02

 .00399934369744

 .00399934369744

 .008531933221217

 .000205094548587

 .00832683867263

 0

 0

 2.05094548587E-05

 2.05094548587E-05

 0

 0

 .00205094548587

 .00205094548587

 0

 0

 0

 0

 0

 0

 0

 0

 1.7022847532687E-03

 0

 0

 0

 0

 0

 .00168177529841

 0

 0

 0

 .00168177529841

 0

 0

 0

 .00168177529841

 0

 0

 .00168177529841

 2.05094548587E-05

 0

 0

 0

 0

 2.05094548587E-05

 2.05094548587E-05

 2.05094548587E-05

 0

 0

 0

 0

 0

 0

 0

 0

 0

 0

 0

 0

 0

 0

 0

 0

 0

 .519176340293375

 .350547602445161

 3.1789655030974E-03

 3.1789655030974E-03

 .00077935928463

 0

 .00235858730875

 0

 2.05094548587E-05

 0

 0

 0

 0

 2.05094548587E-05

 .000840887649206

 0

 0

 .000840887649206

 .000840887649206

 4.2864760654687E-03

 4.2864760654687E-03

 0

 0

 .00426596661061

 0

 0

 2.05094548587E-05

 0

 0

 0

 0

 0

 0

 0

 0

 0

 0

 0

 0

 0

 0

 .00330202223225

 .00330202223225

 .00330202223225

 0

 0

 0

 0

 0

 0

 0

 0

 0

 0

 0

 0

 0

 .321137044177784

 1.92583781122604E-02

 0

 .0153205627794

 .00135362402067

 .00180483202756

 0

 0

 0

 0

 0

 4.10189097174E-05

 .000738340374913

 0

 0

 .024549817465897

 .0197300955741

 .000984453833217

 .00367119241971

 .00016407563887

 .00131260511096

 0

 .00131260511096

 0

 0

 0

 .00842938594692

 .00842938594692

 0

 0

 0

 .192214610936099

 .000184585093728

 .185261905739

 0

 0

 .000410189097174

 .00125107674638

 .00112802001723

 .000143566184011

 0

 .00178432257271

 6.15283645761E-05

 .00198941712129

 0

 0

 .000102547274293

 .000102547274293

 .075269699331354

 .0363427540096

 .0317076172115

 0

 .00123056729152

 0

 .000143566184011

 .000615283645761

 0

 0

 .00432749497518

 0

 .000902416013782

 .002912342589934

 .002912342589934

 0

 .000410189097174

 .00250215349276

 0

 0

 0

 0

 0

 0

 0

 0

 0

 0

 0

 0

 .00135362402067

 0

 0

 .00135362402067

 .00135362402067

 0

 0

 0

 0

 0

 0

 1.35362402067517E-02

 1.35362402067517E-02

 0

 2.05094548587E-05

 0

 0

 0

 0

 0

 .000102547274293

 0

 .0134131834776

 0

 0

 0

 0

 0

 0

 1.8048320275697E-03

 0

 0

 0

 2.05094548587E-05

 0

 0

 0

 2.05094548587E-05

 2.05094548587E-05

 0

 0

 0

 0

 0

 0

 .001784322572711

 0

 0

 .00116903892695

 .00116903892695

 0

 0

 0

 0

 0

 0

 0

 0

 .000615283645761

 0

 .000615283645761

 0

 0

 0

 0

 0

 0

 0

 0

 0

 0

 0

 0

 0

 0

 0

 0

 0

 0

 0

 0

 0

 .166823905820644

 0

 0

 0

 0

 0

 0

 0

 0

 0

 .00557857172156

 0

 0

 .00557857172156

 .00557857172156

 6.24717994996135E-02

 6.24717994996135E-02

 .0137208253005

 0

 0

 .000266622913163

 0

 .0373887362074

 2.05094548587E-05

 0

 0

 0

 0

 0

 0

 0

 0

 0

 2.05094548587E-05

 0

 .000676812010337

 0

 0

 0

 .00352762623569

 .00139464293039

 0

 0

 .000102547274293

 .000984453833217

 .0012920956561

 .00301488986423

 0

 0

 6.15283645761E-05

 0

 0

 0

 0

 0

 0

 0

 0

 0

 0

 0

 0

 0

 0

 0

 0

 0

 0

 0

 0

 0

 .000553755281185

 .000553755281185

 .000553755281185

 0

 0

 0

 0

 .017166413716716

 .017166413716716

 .00730136592969

 0

 .000287132368022

 0

 .000881906558924

 0

 0

 0

 0

 .00869600886008

 0

 0

 0

 0

 0

 0

 .00299438040937

 .00299438040937

 .00299438040937

 .0416957217277

 0

 0

 .0416957217277

 .0416957217277

 .000615283645761

 .000615283645761

 .000615283645761

 1.10751056237174E-02

 4.10189097174E-05

 0

 0

 4.10189097174E-05

 0

 0

 .011034086714

 .011034086714

 0

 0

 0

 0

 0

 0

 0

 0

 0

 0

 0

 0

 0

 0

 .00155871856926

 .00155871856926

 .00155871856926

 0

 0

 0

 0

 0

 2.31141556257607E-02

 1.88071701054387E-02

 .00100496328808

 0

 0

 .0177816973625

 2.05094548587E-05

 0

 .004306985520322

 .000143566184011

 0

 0

 0

 .00324049386767

 .000922925468641

 0

 0

 0

 0

 0

 0

 0

 0

 0

 0

 .01251076746381

 .01251076746381

 .00931129250585

 0

 0

 0

 0

 0

 .00931129250585

 .00931129250585

 0

 0

 0

 0

 0

 0

 0

 0

 0

 0

 0

 0

 0

 0

 0

 0

 0

 .00319947495796

 .00319947495796

 .00319947495796

 0

 0

 0

 0

 0

 0

 0

 0

 0

 0

 0

 0

 0

 0

 0

 0
