# Supplementary figures and images for: The microbiome profiling of fungivorous black tinder fungus beetle Bolitophagus reticulatus reveals the insight into bacterial communities associated with larvae and adults
Source: PeerJ. 2019 May 7;7:e6852. doi: 10.7717/peerj.6852 (PMC6510215; doi:10.7717/peerj.6852)

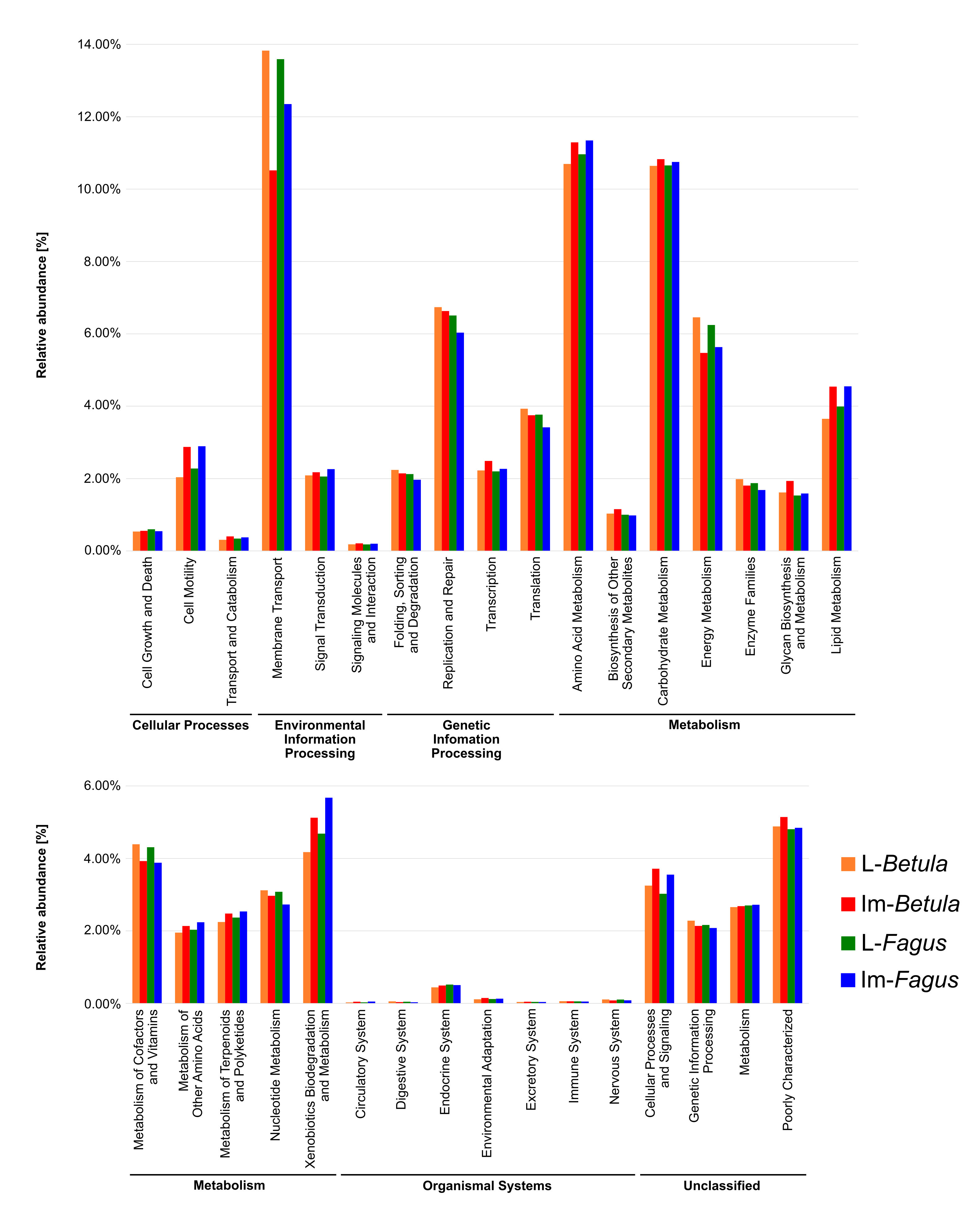

Supplement: Figure S1 — All of the predicted KEGG metabolic pathways are shown at the second hierarchical level and grouped by major functional categorie. [file peerj-07-6852-s004.png]
